# Neuromorphic Optical Tracking and Imaging of Randomly Moving Targets Through Dynamic Dense Scattering Media

**DOI:** 10.1002/advs.77082

**Published:** 2026-08-24

**Authors:** Ning Zhang, Arto Nurmikko

**Affiliations:** ^1^ School of Engineering Brown University Providence Rhode Island USA

**Keywords:** artificial intelligence, artificial neural networ, computer vision, diffuse scattering, energy efficient algorithms, neuromorphic engineering, optical reconstruction, optical trackin, spiking neural network

## Abstract

Imaging and tracking objects moving along random, unpredictable trajectories through dense scattering media remains an open challenge with direct applications in autonomous navigation, underwater robotics, and biomedical sensing. Here we present, to our knowledge, the first end‐to‐end brain‐inspired neuromorphic system that accomplishes both tasks simultaneously. A dynamic vision sensor (DVS) captures photons interacting with dynamic targets while suppressing the static “foggy” scattering background, yielding sparse spike trains. These feed directly into a deep spiking neural network (SNN) with dual modules: an Object Tracking Module for real‐time localization and an Object Reconstruction Module with residual refinement for high‐fidelity spatial recovery. Temporal memory in leaky integrate‐and‐fire neurons lets the network accumulate evidence across time steps, reconstructing consistent target geometry despite substantial motion‐induced variation in spike patterns. We validate the system in transmission and reflection geometries using solid optical phantoms, turbid water, and dynamic fog, with two‐dimensional targets of increasing complexity—MNIST digits, Kanji characters, and birds executing naturalistic trajectories. The system achieves structural similarity indices up to 0.96 and mean squared errors as low as 0.004, with full‐sequence inference completing in under 15 ms. Energy analysis indicates 18‐fold reductions relative to equivalent artificial neural networks, positioning the approach for emerging ultralow‐power neuromorphic hardware.

## Graphical abstract

Vision begins not with images but with change: the retina fires only when something moves, handing the brain a stream of spikes. A neuromorphic imaging system adopts the same strategy—an event sensor that sees like the retina, a spiking network that thinks like the brain—to track and reconstruct fully randomly moving targets through tissue (phantom), turbid water, and dynamic fog.

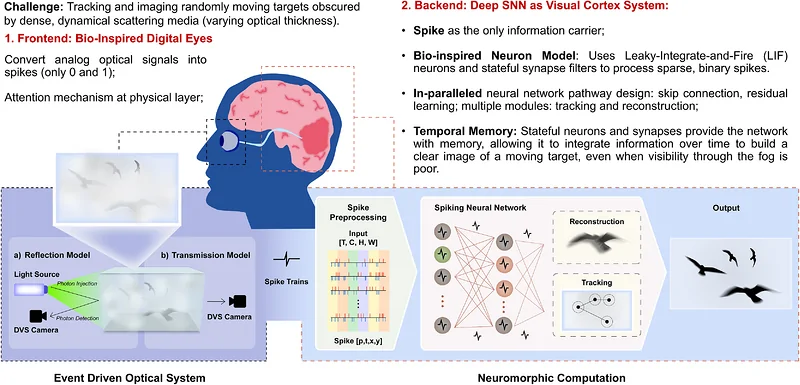

## 1 Introduction

Acquiring high‐resolution images of targets obscured by turbid media is a fundamental challenge in optical imaging, particularly while objects of interest move randomly, along unpredictable trajectories. Whether identifying vehicles navigating in dense fog or vessels moving in turbid underwater environments, isolating dynamical physiological events in biological tissue or communicating via structured light through clouds, the problem is common as it is fundamental. Yet despite much work on optical imaging in turbid media, to our knowledge no existing method can simultaneously track and reconstruct randomly moving objects in highly scattering media.

Recent strategies have exploited advances in hardware and computational methods. Use of time‐of‐flight and time‐gated methods can help isolate targets by exploiting selective depth information [1, 2, 3, 4], while compact LiDAR modules which integrate single‐photon avalanche diode arrays (SPADs) enhance performance [5, 6, 7, 8, 9, 10]. Laser speckle and photon correlation techniques can extract spatial information from statistical signatures in scattered light fields [11, 12, 13, 14, 15, 16, 17, 18] and various optical field manipulations—wavefront shaping, gated holography, ghost imaging, and multiple‐scattering inversion—have demonstrated success under controlled turbidity [19, 20, 21, 22, 23]. Recent work further reviews ghost‐diffraction approaches and demonstrates deep‐learning and diffusion‐prior super‐resolution ghost imaging through complex and even dynamic scattering media [24, 25].On the computational side, convolutional neural networks (CNNs) and related machine learning methods have improved prospects for real‐time imaging [6, 26] using architectures that include hybrid CNN/dense networks such as in lensless imaging through static scattering media, U‐Net segmentation of consecutive speckle frames, and recurrent models fusing temporal dynamics with phase spectra to track targets under controlled motion [27]. The majority of work has dealt with spatially stationary targets in moderately dense media and focused on optimizing systems for image classification or static reconstruction. Further, studies involving moving targets have relied on controlled, predictable trajectories—conditions rather removed from the stochastic motion in many real‐world scenarios. Finally, the computational burden of artificial neural networks typically deployed on power‐intensive GPUs conflicts with the ultralow‐power and low‐latency requirements of mobile platforms such as those required in autonomous vehicles and wearable sensors.

Recently, neuromorphic machine learning has emerged as a different computational paradigm in the field of dynamical optical imaging. In particular, event‐based sensors such as those used in this work, paired with spiking neural networks (SNN) have demonstrated success in trajectory prediction, object localization, optical flow estimation, and gesture recognition. While published SNN models benefit from the natural compatibility between asynchronous sensor output and spike‐based computation to achieve low latency and high energy efficiency [28, 29, 30, 31, 32, 33, 34, 35, 36, 37, 38], in prior work the environments were optically transparent and did not address the challenge of dense, light‐scattering turbid media.

We hypothesize that a fully neuromorphic approach, mimicking human (mammalian) vision, might overcome present limitations in attempting to “see through dense fog.” As a physiological instrument, the eye is particularly attentive in detecting object movements while their images are built by temporal integration in the brain. Crucially, all information from the retina to the visual cortex is transmitted and processed as spikes, enabling operation at very low power and latency. In this paper, we combine a commercial dynamic vision sensor (DVS) [31, 39, 40, 41] as a proxy for the retina with a custom spiking neural network model for the visual cortex. Here, the idea is that a DVS camera responding selectively to temporal intensity changes caused by the target dynamics might also be effective in suppressing the dominant scattering background in turbid media. Importantly, because the DVS camera output is encoded as asynchronous spike trains, salient information can then be transmitted to and processed directly by a spiking neural network (SNN) module without any data processing overhead. We designed an SNN deep learning model to perform object tracking and image reconstruction, here of two‐dimensional images and silhouettes, in parallel while leveraging sparse, event‐driven computation and intrinsic membrane‐potential memory of spiking neurons to achieve low latency and high energy efficiency in an end‐to‐end neuromorphic imaging pipeline.

The principal contributions of this work are threefold. First, temporal‐contrast sensing for turbid media: we demonstrate below that an event sensor, by capturing only temporal intensity changes, suppresses the static scattering background and thereby enables tracking and image reconstruction of randomly moving targets through dense turbid media. Second, a modular SNN for joint tracking and reconstruction: we introduce a spiking neural network with parallel object‐tracking and image‐reconstruction modules that process all information as asynchronous spikes. The SNN architecture exploits sparse, event‐driven computation and the model's inherent ‘statefulness’ (memory) to achieve low latency (< 15 ms) and lower power (estimated 18‐fold less than ANN) on a standard GPU. Third, experimental validation across diverse scattering environments: whether imaging dynamic targets in solid phantoms under reflection and transmission geometry or mimicking turbid underwater or dynamic fog environments on benchtop, our system yields high‐fidelity image reconstruction of randomly moving targets with structural similarity indices (SSIM) ranging from 0.81 to 0.96 as well as accurate trajectory recovery.

## 2 Methods

We next describe the co‐design of an event‐driven optical system and the spiking neural network model by addressing the two subsystems separately. The asynchronous DVS camera as a “front end” to the system provides qualitatively different input data from standard cameras—sparse temporal contrast— which we chose as the tool suitable for isolating dynamic targets in highly light‐scattering environments. The SNN model, in turn, is naturally matched to process the spiking data directly for the dual goal of tracking and imaging randomly moving targets.

**FIGURE 1:**
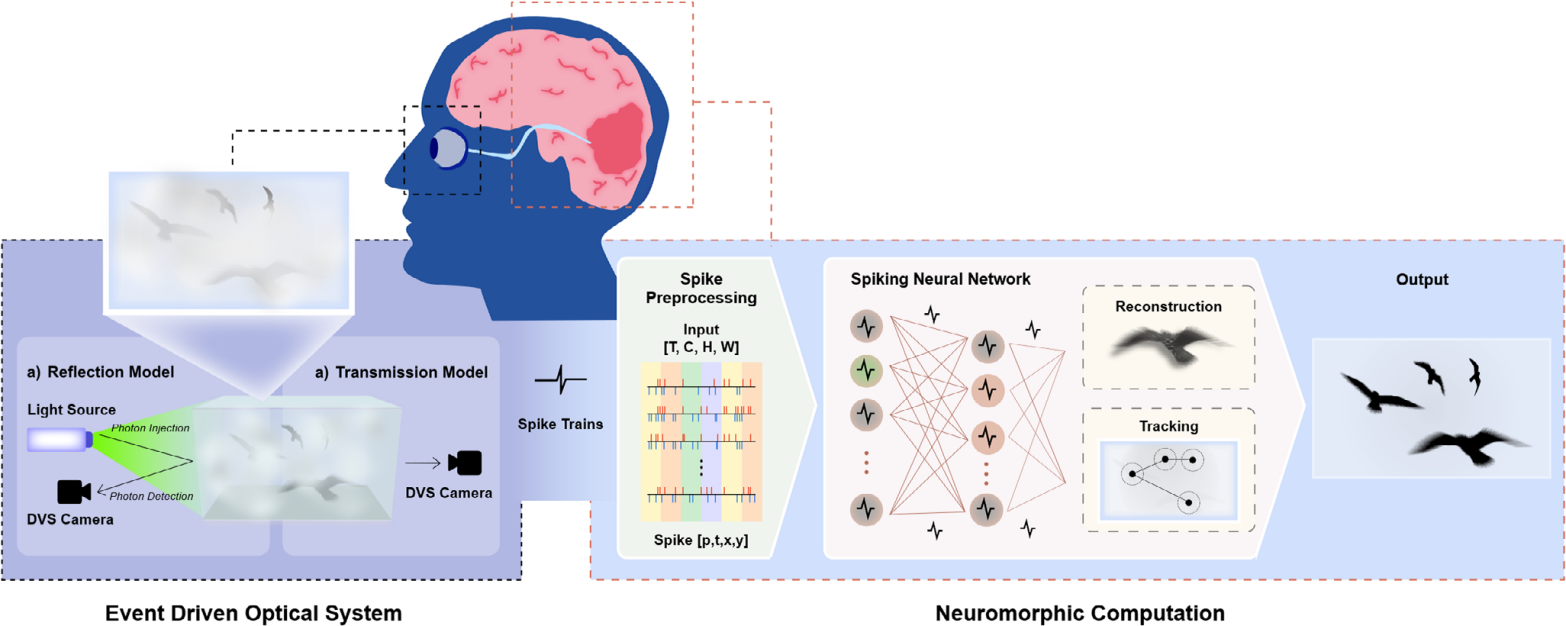
Concept view of end‐to‐end neuromorphic approach for tracking and reconstructing images of dynamic targets obscured by turbid media (cartoon of a randomly moving bird). The direction of data flow shows the integration of the benchtop event‐driven optical subsystem (transmission or reflection mode, left) with a deep spiking neural network (SNN) engine (outline of the neuromorphic computational architecture, right). Asynchronous spike trains generated by the optical subsystem favor those photons which interact with a dynamical target, reducing data overload by the uninformative static background.

Figure 1 shows the concept of a brain‐inspired imaging system. As a proxy for the retina, the event‐sensing dynamic vision camera exploits available technology, whereas the deep spiking neural network (SNN) is custom designed to mimic salient features of the visual cortex. Crucially, all key information in our approach—from scene capture through transmission to processing by the “back end” SNN computing engine—is based solely on asynchronous, spike‐based representation, analogous to action potentials in the nervous system. We reasoned that a spike‐based approach might not only help to improve dynamical imaging in dense turbid media but could also reduce bandwidth and power consumption without compromising high throughput.

### 2.1 Event‐Driven Optical Imaging

**FIGURE 2:**
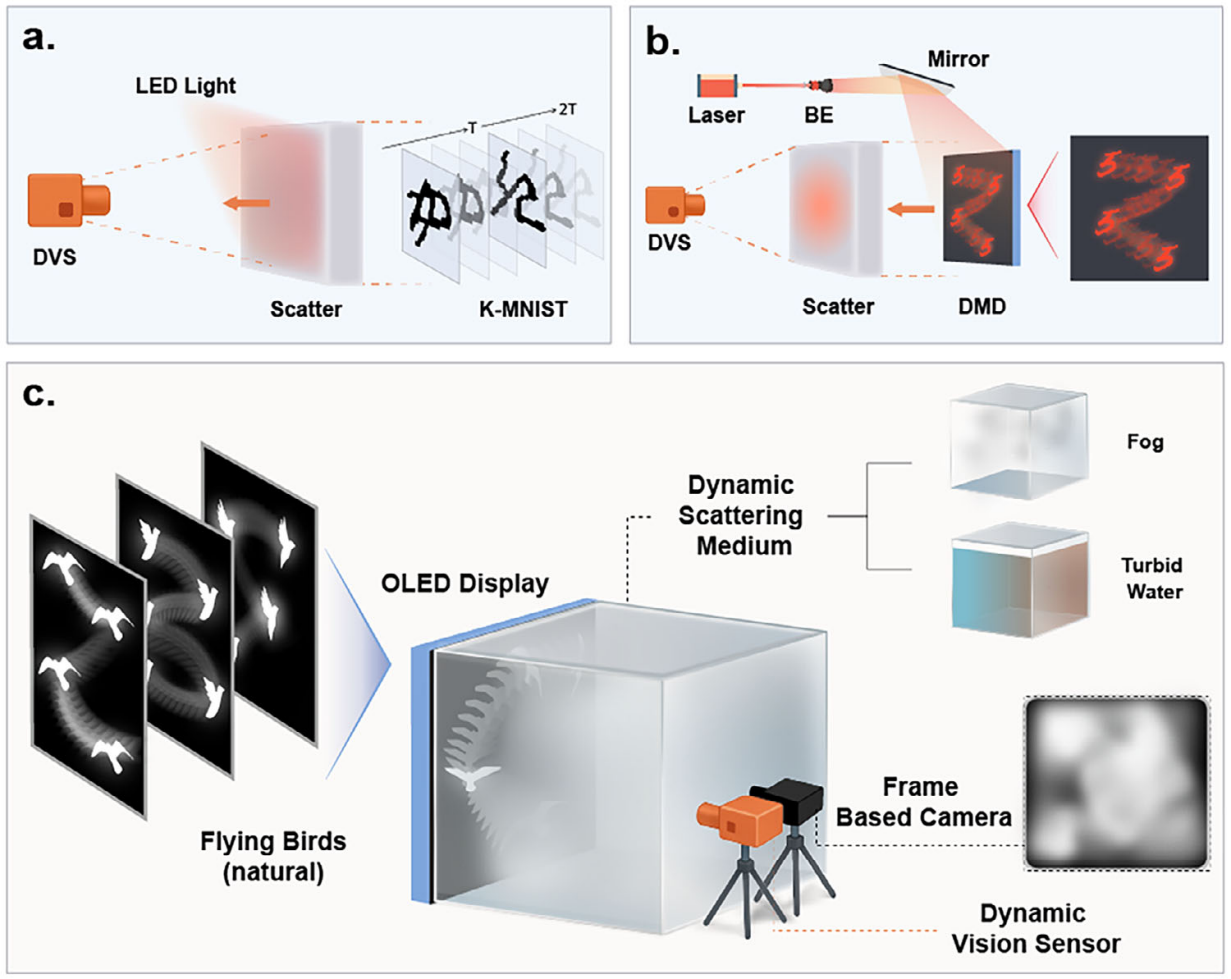
Benchtop optical imaging experiments. (a) Backscattering (reflection) geometry for imaging spatially stationary “flashing” two‐dimnesional targets (K‐MNIST characters) through a slab of solid turbid medium, here using an infrared LED as the source of illumination. (b) Transmission geometry in the case of the solid medium with randomly moving images (MNIST characters) projected through the medium using a beam‐expanded (BE) laser beam incident on a digital micromirror device (DMD) scanner. (c) Transmission geometry in the case of a tank of turbid water or a fog chamber where randomly moving targets (images of birds) were generated by an OLED display and projected through the medium. In each case photons were detected by a dynamic vision sensor (DVS); a frame‐based CMOS camera was used for reference.

Benchtop experiments employed two optical geometries:reflection and transmission, and three projection methods for target images. Our aim was to experiment with a range of illumination conditions and image projection methods to demonstrate the broader utility of the approach toward practical applications. In the reflection mode, an infrared LED (10 mW, 850 nm) illuminated a passive E‐ink display positioned behind the scattering medium (Figure 2a) to generate flashes of *spatially stationary* targets with time‐varying contrast at a 2.5 Hz refresh rate. For randomly moving targets, we used the transmission mode where structured light was projected through scattering media either by a scanning digital micromirror device (DMD) for diffracting laser light (5 mW, 850 nm) at 100 Hz, or by a programmable OLED display projecting white light target images at 120 Hz (Figure 2b,c). Configuration choice (E‐ink, DMD, or OLED) was made depending on the turbid medium type, the target dynamics, and the required field of view. The technique of 2D optical projection in lieu of physical objects is an effective and pragmatic approach used by many authors [15, 16, 18, 26, 27, 42, 43].

The transmitted or reflected (backscattered) light from turbid media was recorded with a DVS camera with a frame‐based CMOS camera as reference for optical alignment. Scattering in each medium was quantified by optical thickness (OT) measured by a transmitted probe laser power as OT = − log (P/P_0_) where P_0_ is the power measured in the absence of the scattering medium, and P is the power in its presence. We also had available parameters such as the scattering coefficient µ_s_ and anisotropy factor g as reported in Section S1 with photon mean free path estimates. For clarity, we use the quantity OT throughout the paper. We point out that in the majority of papers published on imaging in turbid media, there is inadequate quantitative characterization of the degree of opacity, which makes assessment and comparisons between different works ambiguous. Here, three types of scattering media were deployed: (i) solid phantoms of 12 mm thickness (Figure 2a,b), fabricated as SiO_2_ microparticle‐doped silicone with OT ≈ 3.3; (ii) the turbid liquid medium (Figure 2c) was an Intralipid suspension in a 30 cm water tank calibrated to OT ≈ 3.5; and (iii) the vapor medium (Figure 2c) was a fog chamber of 35 cm path length, also exhibiting a mean OT ≈ 4 with temporal fluctuations characterized in Section S2, M4. The turbid water‐tank experiments are presented in the Results section; fog‐chamber experiments are summarized in Section S2. OT values were all measured at 850 nm wavelength.

Event data were acquired using a DVS camera (Prophesee Gen 3.0 EVK, 640 × 480 pixels, 9.6 × 7.2 mm^2^ active area). Unlike frame‐based cameras that sample intensity at fixed intervals, a DVS generates an event whenever the logarithmic intensity at a pixel changes by more than a contrast threshold θ (see more details of DVS in Section S8–S10). Positive events correspond to intensity increases and negative events to decreases. In our experiments, the contrast threshold and bias parameters were tuned to balance event rate and noise, with θ ≈ 12%. Under these settings, the DVS provided a dynamic range of up to 120 dB, microsecond‐level timestamping per event data capture, and low power consumption (< 1 W) with minimal latency to make the camera well suited for real‐time dynamical imaging. We note that because photon transport through scattering media and the sensor's readout electronics are stochastic, there is no deterministic one‐to‐one mapping between the underlying object dynamics and the recorded event stream.

As is common in event‐based processing systems that interface with synchronous computational frameworks, our preprocessing stage for the computing module involved temporal binning of the raw asynchronous DVS events. Events (*x*, *y*, *p*, *t*) where p is the spike polarity, were preprocessed in two stages before inputting to the spiking neural network. First, spatiotemporal denoising filters removed isolated events inconsistent with local activity. Specifically, we applied an activity filter that discarded events without neighbors within a temporal window of 50 ms and a spatial radius of 10 pixels, rejecting low‐probability noise while retaining coherent event clusters associated with target motion or contrast changes. Second, the remaining asynchronous events were temporally binned into *N* tensors of shape [*T*, *C*, *H*, *W*], where *T(= N)* indexes discrete time steps, *C*  =  2 encodes positive and negative polarities, and *H* × *W* corresponds to the camera spatial resolution (640 × 480 pixels). Each bin spanned a duration Δ*t*, hence the total event (sequence) duration was *T*_duration_ =  *N*Δ*t*. For example, in Figure 2b experiment (randomly moving MNIST characters in the solid phantom), we used Δ*t*  =  20 ms and *N*  =  18, providing sufficient temporal resolution to resolve target trajectories while keeping the computational load manageable for training and inference. This discretization is a very common and practical choice in current gradient‐based SNN training frameworks; native neuromorphic hardware such as Intel's Loihi 2 could, in principle, process microsecond‐resolved events without explicit binning.

One asset of the optical hardware of Figure 2 is its simplicity, which should allow future implementation in field‐portable or body‐wearable use when deploying compact components such as low‐power semiconductor laser sources and micro‐optical elements. Low power can be critical if one envisions the concepts in this paper translated to lightweight mobile platforms such as autonomous vehicles.

### 2.2 Deep Spiking Neural Network Model (SNN)

**FIGURE 3:**
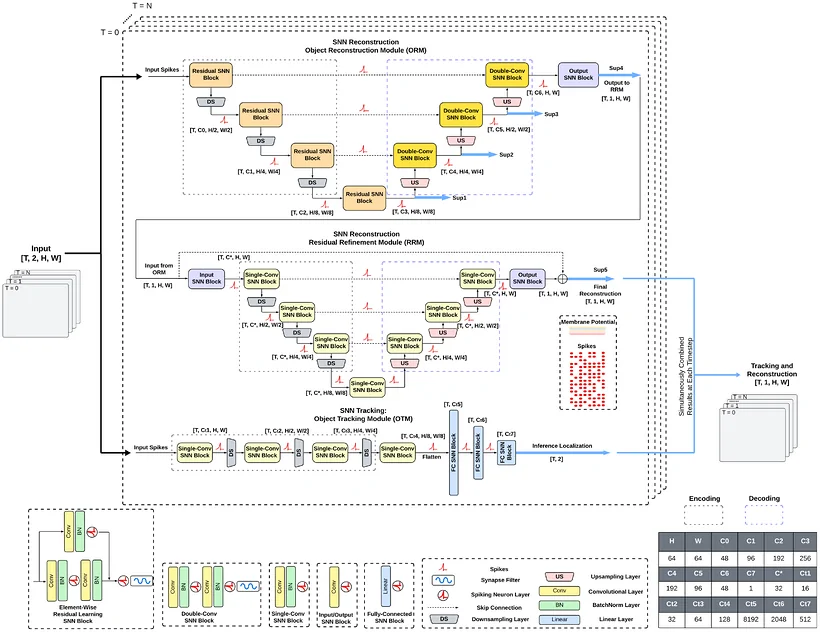
SNN Architecture for Dynamic Object Tracking and Reconstruction in a two‐module approach to simultaneously track and reconstruct dynamic objects obscured by scattering media. The SNN leverages spiking neural computation to process both spatial and temporal features. Input spike trains from the DVS are preprocessed into spiking tensors and processed by an Object Tracking Module (OTM) and an Object Reconstruction Module (ORM). The OTM in the lower part of the large panel in Figure 3 performs object localization, outputting normalized x‐y coordinates of the center of the target at each time step. Its structure consists of an encoding path, which extracts spatial information into latent representations, and a linear mapping path that transforms this data into coordinate predictions. x‐y coordinates are derived from the membrane potential of the last spiking neuron (SN) layer, which resets per time step to handle random object movements and preserve temporal context in the intermediate layers. The ORM, based on a spiking U‐Net‐like framework in the uppermost panel, reconstructs images of objects from input spike trains. The module consists of an encoding path, a decoding path, and skip connections for retaining fine‐grained spatial details. The encoding path progressively reduces spatial dimensions while the decoding path restores spatial resolution using upsampling layers and convolutional SNN blocks. The output from the reconstruction module is input to a Residual Refinement Module (RRM), which improves boundary sharpness and regional consistency by learning residual differences between coarse reconstructions and ground truth. Hybrid loss functions optimize training, with multi‐stage losses applied from the bottleneck to each decoding stage (denoted as "SUP" by blue arrows). Reconstruction loss combines Binary Cross‐Entropy (BCE) for pixel accuracy, structural similarity index (SSIM) for patch coherence, and Intersection over Union (IoU) for map‐level coverage. Tracking loss uses Mean Square Error (MSE) to refine predicted object center coordinates. Structures of the various SNN blocks are shown at the bottom of the figure, including element‐wise residual learning SNN blocks, stateful synapse filters, and others. The architecture incorporates nearest neighbor interpolation upsampling layers and max‐pooling downsampling layers to efficiently manage spatial transformations. Spiking neurons in all layers encode spatiotemporal information into membrane potentials and spike outputs, aligning with the event‐driven nature of the input data. The [640,480] raw input was preprocessed into [64,64] in height and width, as shown in the table at bottom right.

Here we outline the design rationale, structure, and operating regimes of the SNN model of Figure 3, designed specifically for optical imaging in turbid media. Algorithm details in the architecture, such as layer definitions, supervision weights, optimizer settings, and train/test datasets, can be found in the Appendix (S4, S9 and S11).

In brief, we developed a fully neuromorphic pipeline that converts the raw, asynchronous DVS events into time‐resolved spike tensors and performs both tracking and image reconstruction functions in parallel. Following the discretization of the input spike trains into [T, C, H, W] tensors, the data branches simultaneously to an Object Tracking Module (OTM) and an Object Reconstruction Module (ORM); the ORM output is further refined by a Residual Refinement Module (RRM). This division reflects the different temporal demands of the dual task: coordinate localization for target tracking can be lightweight and low‐latency, whereas high‐fidelity image reconstruction benefits from deeper temporal integration. Both branches process the same spiking input with outputs fused at each time step to ensure synchronization of trajectories and reconstructed frames. Splitting reconstruction into the ORM and the RRM, rather than using a single integrated network, is a deliberate coarse‐to‐fine strategy motivated both by the physics of imaging through dense scattering media and by the goal of optimizing the SNN engine under these challenging conditions, as detailed below and in Section S4.

To maintain temporal evidence in the dynamics of spiking neurons and their synapses, we used leaky integrate‐and‐fire (LIF) neurons to maintain membrane potentials that carry short‐term memory; synapse filters make the synapses stateful, further enhancing temporal retention. Training across multiple time steps rather than single snapshots allows the model to separate target movements that might otherwise produce similar instantaneous spike patterns, and to remain robust when the same target generates different spiking patterns due to changes in random trajectory (direction, speed, and acceleration). This temporal memory also helps distinguish different targets producing similar spikes at any given time by leveraging memory from other times as motion evolves.

The OTM (lower part of Figure 3) is intentionally compact: a downsampling encoder of convolutional SNN blocks followed by a shallow fully‐connected SNN head that maps the latent features to coordinates (x, y). A key design feature is that only the final spiking layer resets at each time step, while all earlier layers preserve their membrane states. A neuron's membrane potential accumulates readily from the received input and decays only through leakage or firing. The reset policy prevents accumulated high potentials from introducing (biasing) sudden coordinate jumps while maintaining hidden layers’ temporal context for stable tracking over random trajectories—and keeps tracking latency low with good accuracy. The tracking loss is quantified as Mean Squared Error (MSE) between the predicted center of gravity and ground truth.

For image reconstruction, the ORM (upper part of Figure 3) follows a spiking U‐Net‐like encoder–decoder architecture with skip connections to recover spatial detail. The subsequent Residual Refinement Module (RRM) is a much smaller spiking encoder–decoder that learns a residual correction on top of the ORM output. The reason for splitting reconstruction this way follows from the photon budget in a dense scattering medium: because the number of target‐interacting photons is intrinsically limited, the coarse silhouette of the target survives transport with higher signal‐to‐noise than its finer spatial features. Recovering the target is therefore naturally a two‐step problem—first recover the coarse spatial layout from the higher‐SNR component of the signal, then restore the finer detail that scattering and noise have masked—rather than asking a single network to deliver the final map directly. Because the RRM operates only on a low‐dimensional residual, it stays shallow and inexpensive and preserves the energy advantage of the SNN reported in Table S1. The physical rationale is detailed in Section S4, and the quantitative benefit is given by the ablation study in Table S2. This coarse‐to‐fine structure incorporates multi‐stage supervision (SUP): intermediate predictions from five decoder stages are upsampled and compared to the ground truth, stabilizing gradients and encouraging hierarchical feature learning. Reconstruction is optimized by a hybrid loss from pixel‐level to overall similarity comparison. We chose binary cross‐entropy (BCE) for pixel accuracy, intersection over union (IoU) for spatial overlap, and structural similarity index (SSIM) for perceptual structure. The three reconstruction terms are equally weighted (α = β = γ = 1) unless otherwise noted.

The two modules were trained separately with their respective losses without cross‐module backpropagation—each branch optimized its own task. At the inference stage, the two modules are fused to process the same input simultaneously. In tasks with spatially static positions (e.g., reflection experiments with flashing images), we can disable the OTM and train/use the ORM/RRM alone to reduce computation, while in tasks that involve random motion (transmission experiments with randomly moving targets) both branches run in parallel for synchronized outputs.

A remark on event discretization: converting the raw microsecond‐scale DVS camera output stream into fixed‐step tensors is a necessity for current backpropagation‐based spiking training frameworks and serves as a rate‐control buffer when the object scene dynamics drive very high event rates.

## 3 Results

Experimental results are presented for different case studies, progressively increasing the level of challenge in imaging through dense turbid media: (i) spatially stationary K‐MNIST(Kanji) characters with time‐varying optical contrast, imaged through the solid turbid phantom in reflection geometry; (ii) randomly moving MNIST digits behind the solid phantom but now in transmission geometry; (iii) 2D silhouettes of species of birds executing naturalistic random flight movements recorded in transmission through a tank of turbid water; and (iv) randomly moving targets in a dynamically drifting fog, where the optical thickness fluctuated during acquisition (summarized in Section S2).

### 3.1 Image Reconstruction of Spatially Stationary Time‐Varying Targets (Solid Phantom, Reflection)

**FIGURE 4:**
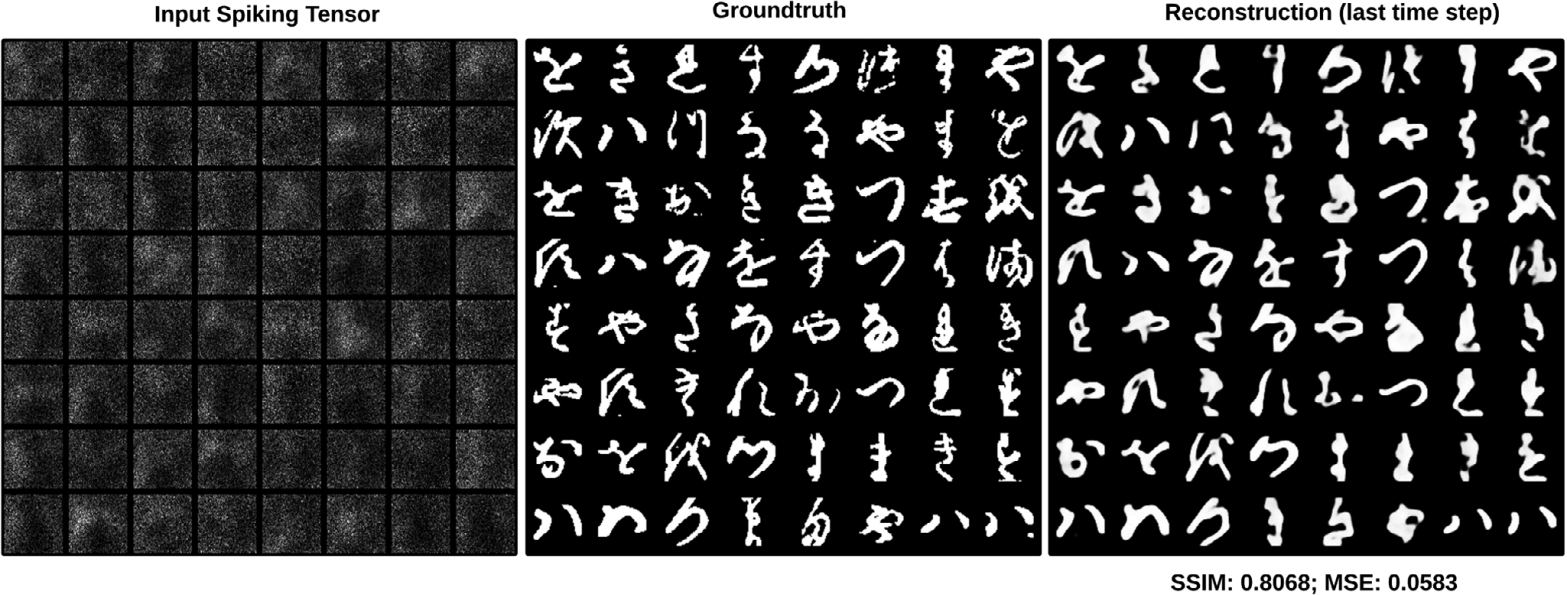
Results of experiments conducted in reflection (backscattering) geometry using Kanji characters as targets. Each character was dynamically presented as a flash emerging and disappearing once per object on an E‐ink display to mimic optically dynamic but spatially stationary targets. Although the raw output from the DVS camera is unrecognizable (left panel), the neuromorphic imaging method produced reconstructed images that were clear and unambiguous. Training and testing used 25 000 and 3000 characters, respectively, and quantitative evaluation on the testing dataset yielded SSIM = 0.8080, PSNR = 12.66, MSE = 0.058, and MAE = 0.0716.

Figure 4 summarizes experiments conducted in reflection (backscattering) geometry using Kanji characters as spatially fixed but optically time‐varying targets. Each character was flashed once (to appear/disappear) by using a programmable E‐ink display (eink.com) placed behind a slab of solid silicone–SiO_2_ microparticle phantom (see Methods and Figure 2a), the scene illuminated by an infrared LED at 850 nm.

In reflection mode, photons travel through the turbid medium twice, increasing the effective optical path and optical thickness. For the phantom used here, the optical thickness was OT ≈ 6.6, corresponding to ≈144 photon mean free paths (MFPs), rendering the raw DVS data unrecognizable; similarly, not even a hint of the underlying Kanji characters was visible to the naked eye or to a regular CMOS camera. Demonstrating the performance of the neuromorphic combination of event detection and the SNN decoding, we achieve clear reconstructions (Figure 4 compares the reconstruction against the ground truth). We used 25 000 characters for training and 3000 for testing, each event sequence discretized into T = 18 time steps. Across the test set, the reconstruction achieved an SSIM = 0.8080, PSNR = 12.66, MSE = 0.058, and MAE = 0.0716, demonstrating the system's capability for fidelity imaging in this double‐pass optical configuration.

Some of the reconstructions in Figure 4 show inaccuracies, particularly those characters with fine strokes or high stroke density. Object‐specific photons were strongly attenuated in the double‐pass geometry, and the reflection geometry added background noise photons from phantom surface backscatter. Complex characters with thin features (strokes) generate fewer threshold‐crossing intensity changes in the DVS camera, reducing useful event information. Nonetheless, even in such cases of sparse, low–signal‐to‐noise event streams, the system is able to extract meaningful spatiotemporal patterns. Videos of reconstruction are shown in Video S2.

### 3.2 Image Reconstruction and Tracking of Randomly Moving Targets (Solid Phantom, Transmission)

We next consider the case of randomly moving targets as viewed in transmission mode through the same calibrated silicone–SiO_2_ phantom (OT ≈ 3.3) (Figure 2b). Here the targets were MNIST digits behind the phantom, which executed random x–y trajectories; the event formation was dominated by edge motion under strong scattering blur. The kinematics were piecewise linear, approximating a random walk (Figure 7b, left); for smooth random movement, see the next section. Structured light was projected by a DMD at 100 Hz; note that the DVS timestamps events with microsecond precision, more than sufficient to capture the intensity changes induced by target motion.

**FIGURE 5:**
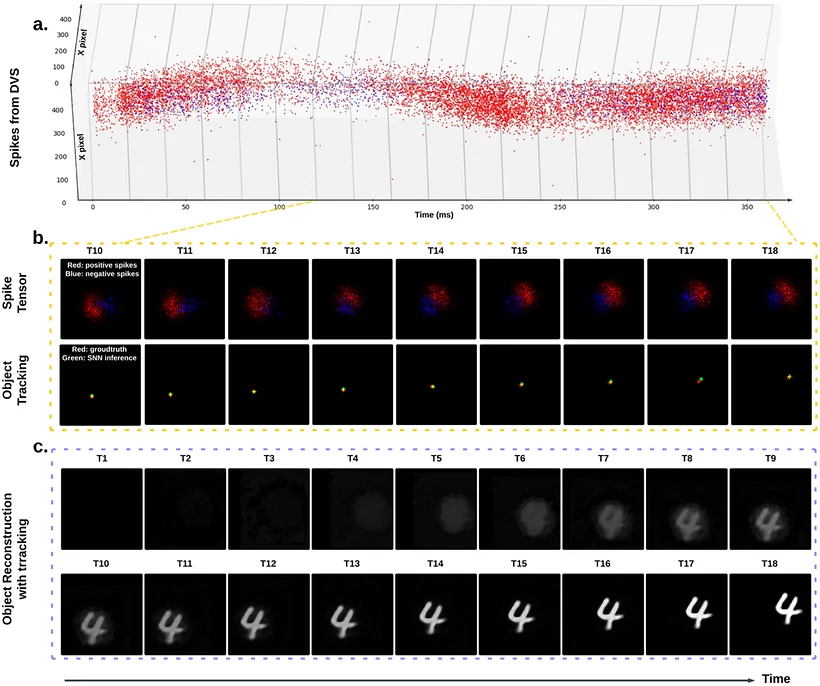
Neuromorphic image reconstruction of a moving object invisible to the naked eye in dense scattering media. (a, b) Data and results for an MNIST digit '4' moving randomly behind the scattering medium as an x,y,t plot. (a) Sample of raw spiking data from the DVS camera. (b) Upper panel: Spiking tensor derived from the preprocessed spikes, represented as [T, C, H, W]. Lower panel: Object tracking, obtained from the OTM, illustrates the spiking neural network (SNN) inference (green crosses) alongside the ground truth positions (red crosses) of the randomly moving target. (c) Target image reconstruction with tracking is the combined result from OTM and ORM at each time step. During a total of 18 time steps the image of digit ‘4’ emerges until clearly and fully recognizable (here snapshots from a Video S1). The average structural similarity index measure (SSIM) and mean square error (MSE) between reconstruction and ground truth are 0.9568 and 0.0108, repectively.

Figure 5 and the Video S1 show an MNIST digit ‘4’ moving randomly behind the scattering medium. Panel (a) illustrates the raw spikes recorded across the 480 × 480 (cropped from 640 × 480) DVS camera pixels displayed as an x‐y‐t space‐time plot. Positive (red) and negative (blue) spikes represent respective increases and decreases in detected pixel‐level light intensity. Panel (b, upper) shows these raw events preprocessed into spiking tensors in the format [T, C, H, W], where T = 18 and C = 2 (polarity channels).

Object tracking by the OTM (lower part of Figure 5b) is shown as inferred object position (green crosses) when compared against the ground truth positions (red crosses). Note that the tracking module localizes the target accurately before reconstruction has converged because it solves a simpler problem—predicting two scalar coordinates rather than a full image. The DVS's motion sensitivity concentrates events near moving objects, providing strong spatial cues even from limited data, and the OTM's streamlined architecture (aggressive downsampling, compact output head) accelerates convergence. Ground‐truth positions and target images were logged at their point of generation before being sent to the DMD for display.

Figure 5c shows the reconstructed images (integrated outputs of the OTM and ORM modules) from time steps T1 to T18. During initial time steps (T1 to T6), the reconstruction appears blurred and lacks discernible shapes because the SNN has received limited spatiotemporal information: LIF membrane potentials are still evolving from sparse input. As additional steps are processed, stateful neurons integrate accumulating events, building a progressively more complete representation. By mid‐sequence (around T7–T12), the digit becomes recognizable; late steps refine boundaries at diminishing returns. The full time evolution of reconstruction is shown in Video S1. The accuracy (measured as SSIM) increases steeply from T = 1 to approximately T = 10, then plateaus; our choice of T = 18 provided an effective balance between quality and latency for the 360 ms event segments used here. The behavior is reminiscent of the human visual process, where temporal memory plays a role in object recognition as the brain builds a recognizable image of a randomly moving target in conditions of weak ambient light. Here, the SNN algorithm leverages its built‐in temporal memory, utilizing early‐stage input data to progressively refine its understanding and recognition of the target.

**FIGURE 6:**
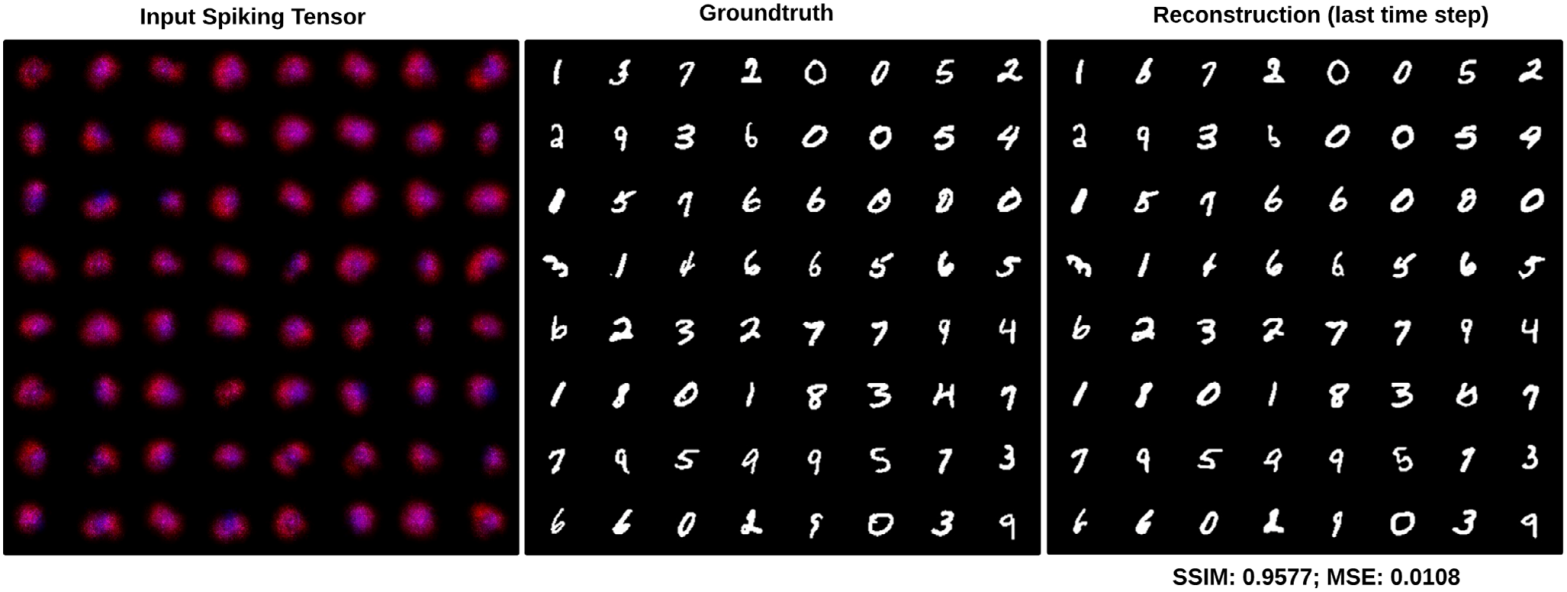
Summary of reconstruction results obtained in transmission geometry for randomly selected digits from the MNIST set, displayed as an 8 × 8 array where each image represents a distinct target. The figure compares the raw spiking data recorded by the DVS camera through the phantom (left), the “ground truth” images (middle), and the final SNN‐generated reconstructions (right). The raw data shows the temporally accumulated spike tensor across all time steps, reflecting the random motion trajectories with blurriness caused by the turbid medium. The reconstructed image array shows the target at the final time step. Quantitative evaluation yields SSIM = 0.9577, PSNR = 20.09, MSE = 0.0108, and MAE = 0.0119. Note that the size of images for ground truth and reconstruction is magnified for visualization purposes relative to the spiking tensor. Time‐lapse videos of image reconstructions can be found in the Video S1.

Figure 6 summarizes the experiments by demonstrating consistency across the full test set where 64 randomly selected digits are displayed as an 8 × 8 array, each having executed a distinct random piecewise trajectory. The figure compares the accumulated spike tensor (left), ground truth (center), and final reconstruction (right). While Figure 5 illustrated the temporal evolution for a single target, Figure 6 affirms that high‐fidelity reconstruction holds across all ten digit classes and previously unseen unique handwritten digits and their random trajectories (held‐out test instances).

We used 10 000 characters for training and 2000 for testing. On an NVIDIA RTX 3090, training required approximately 6 min per epoch; the network inference time was <15 ms for a complete 18‐step sequence—performance which can be expected to improve on dedicated neuromorphic hardware. This is the network inference latency only: it excludes the event‐acquisition window (≈360 ms here, 18 × 20 ms; varies depending on the signal quality and object's moving speed) and the offline preprocessing. The present system therefore operates in post‐processing mode, with overall latency set by how long the DVS must observe the scene rather than by the network (Section S9). Across the test set, the system achieved SSIM = 0.9577, PSNR = 20.09, MSE = 0.0108, and MAE = 0.0119.

### 3.3 Image Reconstruction and Tracking of Complex Targets (Turbid Water, Transmission)

**FIGURE 7:**
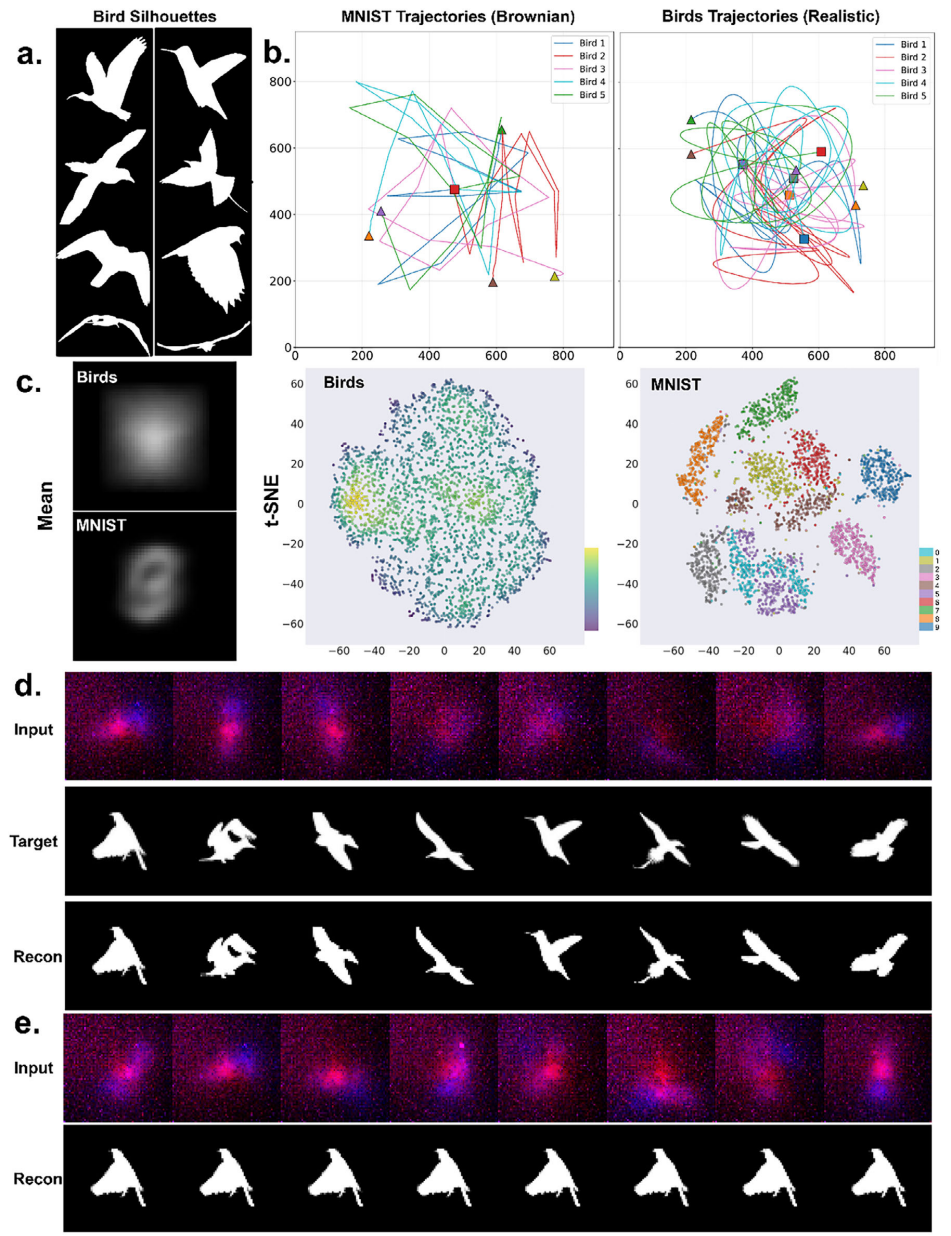
Image reconstruction of complex "flying bird" targets with naturalistic motion trajectories in turbid water. (a) Projection targets: silhouettes of birds in flight, used as the displayed targets in these experiments. (b) Comparison of motion profiles: Whereas the MNIST experiments (Figures 5 and 6) utilized Brownian‐like random motion (left), these experiments employed curvilinear trajectories with varying speed and acceleration covering the full field of view (right). (c) Left: mean image (randomly sampled 500 images) for the Birds and MNIST datasets, showing how the Birds shapes are more complex and widely spread. Right: t‐SNE visualization of feature distributions for both datasets (randomly sampled 3638 images). Unlike the digit datasets, which show distinct clustering by class, the bird dataset features are widely distributed, indicating higher complexity and variance. (d) Reconstruction results for different bird species. From top to bottom: input raw spike tensors (integrated for visualization), ground truth targets, and SNN image reconstructions. (e) Robustness to motion variance: input and reconstruction results for the same bird target moving along different random trajectories. In spite of significant variance in the raw spike input due to motion direction and speed, the SNN was consistently able to recognize and reconstruct the correct bird profile. Metrics: the system achieved a mean SSIM of 0.9593 and PSNR of 28.53 dB across the test set (OT ≈ 3.5).

To further challenge the capability of our neuromorphic approach, we conducted experiments using randomly moving targets of higher geometric complexity in two different turbid environments. Silhouettes of different species of flying birds derived from natural images were projected through a water tank of 30 cm path length by an OLED display unit and the optical medium was rendered turbid by diluted Intralipid solution (OT ≈ 3.5) to simulate a dense underwater scattering environment. In contrast to the stochastic, Brownian‐like piecewise trajectories (Figure 7b, left), we now programmed the birds to execute “natural” flight trajectories characterized by smooth curvilinear paths, varying velocities, and fluctuating accelerations that traversed the full field of view (Figure 7b, right). This introduces a non‐trivial complication inherent to event‐based sensing: because DVS camera output is a derivative of intensity change, the same object generates vastly different spatiotemporal spike patterns depending on its kinematic profile (direction, speed, and acceleration). Yet the problem is closer to many real‐world scenarios such as an autonomous underwater vehicle operating in a dynamic optical environment or a drone navigating in fog, etc.

These experiments also pushed the temporal processing capabilities of our SNN model. Since the DVS is a motion‐sensitive sensor, a slowly gliding bird (or an underwater target) generates a sparse, localized spike cloud, whereas the same target accelerating through a turn generates a dense, distributed torrent of events. Consequently, the neural network cannot rely on a static template matching strategy; it must possess robust temporal memory to integrate diverse spiking information over time in order to converge on a solution. One might view the process being analogous to a hunter spotting moving prey in a dense forest or in fog: a single glimpse is insufficient, but in accumulating visual evidence over time (∼seconds) the brain is able to resolve the ambiguity in the target's shape and form. Our SNN model strives to emulate such biological information accumulation through the stateful membrane potentials of the LIF neurons, effectively building a short‐term working memory that stabilizes the reconstruction despite the chaotic nature of the input spikes. This capability is highlighted in Figure 7e, where the system successfully reconstructs the same image (bird) despite the target moving along a completely different set of trajectories, thereby demonstrating that our model learns the invariant spatial features of an object rather than merely memorizing specific motion‐induced spike sequences. Videos of reconstruction are shown in Video S3.

We utilized a dataset of 50 distinct bird shapes (derived from natural images of flying birds in a publicly available dataset [44]), each executing 100 unique, randomly generated natural trajectories (totaling 5000 sequences split into 4200 for training and 800 for testing). To visualize the complexity of the task, we employed t‐Distributed Stochastic Neighbor Embedding (t‐SNE) analysis on the object feature maps (Figure 7c). While the MNIST datasets exhibit well‐separated clustering—indicating that digits share strong intra‐class similarities—the features in the ‘bird’ dataset are widely dispersed without obvious clusters. The lack of clustering suggests that the feature space for naturalistic targets is more complex and non‐convex; i.e., the features are distributed across a continuous manifold rather than discrete categories. Despite this complexity, where the scattering medium blurs spatial details and the variable motion complicates temporal encoding, our neuromorphic approach yielded high‐fidelity reconstruction (Figure 7d). Quantitative evaluation on the test set yielded a mean SSIM of 0.9593, MSE of 0.004376, MAE of 0.0092, and PSNR of 28.53 dB. The metrics indicate that the system effectively filters out the “noise” of the random trajectories to recover the “signature” of the object geometry, demonstrating robustness required for deployment in unconstrained, dynamical environments.

## 4 Discussion

This work demonstrates an end‐to‐end neuromorphic imaging system that can both track and reconstruct dynamic targets of varying image complexity that are visually unrecognizable when obscured by dense solid, liquid, or vapor scattering media. The asynchronous spike‐generating DVS camera preferentially extracts target‐informing photons from static background scatter, while the SNN uses the same sparse spike stream to perform the dual task of tracking and image reconstruction in parallel (according to the model of Figure 3). The human vision‐inspired design couples sensing and computing in contrast to prior research on “imaging‐through‐scatter” based on frame cameras and CNNs [19, 20, 21, 22, 23, 26, 45, 46, 47]. Our approach does leverage earlier event camera and neuromorphic imaging work in optically fully transparent media, which, however, has focused on specific tasks such as trajectory prediction or gesture recognition rather than imaging.

A major asset of the neuromorphic system is its temporal processing capability. Rather than processing isolated snapshots, the SNN is trained and run on sequences of binned events that represent a complete dynamic episode for a target, typically spanning hundreds of milliseconds of real time (Figures 5, 6, 7). As described in Methods, the stateful LIF neurons and synapse filters let the network integrate information across the full sequence and link successive time steps, stabilizing tracking and reconstruction despite the spike‐pattern variability that random trajectories produce. The upper temporal bandwidth that the pipeline can address is set by three independent factors—the microsecond event latency of the DVS, the SNN time‐step Δt, and the total sequence duration—and within these bounds the system is matched to atmospheric, underwater, and biological scattering processes whose dominant fluctuations lie in the millihertz‐to‐kilohertz range.

The system relies on a separation of timescales between target motion and fluctuations of the scattering medium. In the regimes demonstrated here, the medium varies slowly (for example, fog OT drift below ≈0.013 Hz) and is suppressed by the DVS, while moving targets generate the events used for reconstruction. When the medium's decorrelation rate rises—as in living tissue or strongly turbulent flow—the background itself produces events that raise the effective noise floor and, once they overlap the target's temporal band, can no longer be separated by temporal contrast; in that regime the present DVS–SNN scheme would require re‐tuning and re‐training and ultimately reaches a physical limit. Conversely, under coherent illumination such fast fluctuations carry information, and an event‐based approach could be reconfigured to exploit rather than suppress them.

Across reflection and transmission geometries, from solid phantoms to turbid water and dynamic fog (Figures 4, 5, 6, 7 and Section S2), our method achieves structural similarity indices in the range 0.81–0.96 in image reconstruction with accurate trajectory recovery. The asynchronous DVS front end consumes only tens of milliwatts (∼28 mW) while providing microsecond timing (> 10 kHz effective frame rate), in contrast to high‐speed CMOS cameras (such as 20–40 W consumption in 1000–6400 fps Photron FASTCAM Mini) and mobile CMOS sensors (e.g. 0.5–0.9 W for smartphone CMOS sensors during 4K capture).

Perhaps even more important for practical applications is the estimated 18‐fold reduction in computational energy of the SNN module when compared with standard artificial neural networks (ANNs) (see Section S3). The SNN's computational efficiency comes from operating on sparse DVS spike streams rather than dense frames. Only a fraction of neurons (12% to 20% in our experiments) is active at any time. Compared to dense ANN/CNN models operating on frames, spike‐based processing replaces most multiply–accumulate operations (MAC) with cheaper accumulate operations (AC) on binary spikes, leading to the estimates reported in Table S1 in the Section S3 even when both models are simulated on the same GPU. Altogether, the system offers orders‐of‐magnitude savings in both sensing and inference with a promise for low‐power, mobile, low‐latency sensing in dense scattering environments, particularly once emerging high‐performance neuromorphic hardware is commercially available [48].

Presently available commercial event cameras do have limitations. As noted in Methods, the DVS contrast threshold (≈12% here, 25% nominal; reduced below 2% in recent prototypes [49]) sets a practical lower bound on the contrast changes and motion speeds for which reliable event streams can be acquired. We therefore operated in regimes where target motion and structured‐light modulation produced sufficient event rates for both tracking and reconstruction, while leaving systematic mapping of reconstruction fidelity vs. contrast and speed for future work. Furthermore, imaging through turbid media under low‐light conditions is difficult with a standalone DVS camera, as these sensors rely on conventional photodiodes whose sensitivity degrades as ambient illumination approaches the single‐digit lux range. To extend the DVS to the low‐light range, we have begun exploring a tandem image intensifier—DVS arrangement, while falling beyond the scope of the present paper, we have been able to demonstrate fully neuromorphic imaging operation at light levels down to ∼0.1 lux. We also note the emerging single‐photon avalanche diode (SPAD) array technology and recent work on dual camera SPAD–DVS data fusion albeit in transparent media (air) [31].

Our use of a frame‐based camera in this work was limited to a baseline reference for alignment (for example, monitoring the projected patterns with/without turbid media) rather than as a comparison imaging pipeline. A direct comparison between a frame camera‐ANN and our DVS‐SNN system is complicated and ambiguous because the sensing and processing chains are optimized for different operating points [31]. Frame‐based methods attempting dynamical imaging through turbid media must operate at high frame rates, but this comes at the cost of significantly higher data bandwidth, increased power consumption, and substantial computational load to process continuous frame streams, altogether making real‐time reconstruction difficult for fast‐moving objects. In contrast, our low‐power, low‐bandwidth DVS‐SNN system is tailored to sparse, temporally coded data and low‐power operation, with computation concentrated only on dynamical events. We have compared qualitatively our work with that in recent literature (see link to SOTA analysis at Section S12)

As an additional comment on experimental optical work in turbid media using frame‐based cameras, much of the existing literature uses simple diffusers such as ground glass and rarely quantifies the scattering properties of the medium. Our experiments are grounded in measured optical thickness OT (and photon mean free path) in calibrated solid silicone phantoms, in a turbid water tank, and in a fog chamber.

As for robustness of the SNN model, we note that in the fog chamber experiments the OT fluctuated during acquisition, yet the system maintained stable tracking and reconstruction of moving targets without retraining for each instantaneous OT value (Section S2). This indicates that, at least within a certain range of scattering strengths (optical thickness) in a given medium, the neuromorphic imaging pipeline tolerates moderate temporal variations in turbidity. The largest optical thickness we have demonstrated is OT ≈ 6.6 (≈ 144 MFPs) in the reflection‐mode phantom, beyond which the addressable OT is set by the budget of target‐interacting photons that survive transport and reach the DVS, falling approximately as exp(−OT). Further analyses can be viewed in Section S14. Systematic sweeps over phantom thicknesses or scattering coefficients were not performed here, and a full characterization of robustness across a broader range of calibrated media remains an important direction for future work.

More generally, because the DVS responds to temporal contrast rather than absolute intensity, increases in optical density within given experimental conditions primarily reduce the signal‐to‐noise ratio of the events rather than altering the basic mechanism. In Figures 4, 5, 6, 7, targets with sufficient intrinsic features and motions generate event streams that the SNN could decode reliably across the tested media and OT range. However, we did not attempt to define a single minimum velocity or contrast threshold; these quantities depend jointly on object contrast, illumination condition, scattering strength, object size, and DVS bias settings and would require dedicated studies. The field of view is set by the DVS sensor area, the lens focal length, and the working distance; it scales linearly with working distance and inversely with focal length, so meter‐scale stand‐off scenes and close‐range biomedical surface imaging are immediately accessible by changing the optics with no algorithmic change.

On the other hand, static targets, i.e., targets whose optical properties do not change over time, produce no events beyond sensor noise and as such cannot be reconstructed by the present system. For very slow motions, accumulated events within a practical integration window can be too sparse for high‐fidelity reconstruction, particularly in reflection geometry where double‐pass scattering further reduces signal‐to‐noise ratio. In such cases, adapting an event‐driven approach would likely require inducing artificial relative motion (for example, by scanning the illumination or sensor) or integrating complementary modalities such as SPAD cameras to recover intensity information when intrinsic dynamics are weak.

Finally, the present implementation reconstructs binary profiles rather than grayscale textures. Because the DVS encodes temporal contrast, internal intensity variations that do not cross the event threshold are not preserved. A binary representation is adequate for the experimental targets considered here (characters and bird silhouettes; Figures 4, 5, 6, 7) but would be insufficient for applications that rely on fine grayscale features. This binary limitation is set by the scattering‐limited target information and the supervised prior rather than by the DVS itself: in optically transparent media (absence of scattering), temporal‐contrast events alone can support rich‐texture reconstruction [40, 50, 51] but, under dense scattering, the recovery of fine texture is severely ill‐posed and would require substantially larger training data and generative priors. Grayscale information can in principle be recovered from the events themselves by temporally integrating the event stream to estimate absolute intensity [50, 51], or by fusing the DVS with an additional frame‐based camera. With advances in event sensor technology with lower, better‐controlled contrast thresholds can further improve the access to gray scale in such a fusion approach, albeit at the cost of additional computation, power, and latency. Similarly, extending the approach to multiple randomly moving targets each with its distinct trajectory will require architectural modifications such as parallel processing streams or attention‐like mechanisms to separate overlapping event clusters and more complex training data. The present demonstration applies to targets moving by translation in the image plane; extending the system to reconstruct rotating or flipped targets is a natural next step which can be pursued within the same architecture by augmenting the training data with such added degrees of freedom of target motion. The present system images two‐dimensional targets at a fixed range and is not scale‐invariant; resolving targets that move in depth will require means of depth‐resolved sensing. For example, we are exploring time‐of‐flight gating and binocular configurations of event cameras as more advanced means to extend the tracking‐imaging approach to dynamic three‐dimensional targets ‐ in the presence of dense scattering media.

## 5 Conclusion

We have shown that an event sensing end‐to‐end neuromorphic optical system that uses spikes as the sole unit of information can successfully address the problem of imaging and tracking dynamical and randomly moving targets hidden by dense scattering media. The potential application space is broad. Beyond the phantoms, turbid water, and dynamic fog considered here, similar neuromorphic pipelines could be adapted to head‐mounted or drone‐based systems for fire rescue, identification of vehicles/pedestrians in dense fog, and imaging of dynamical biological targets. In free‐space optical communication and non‐line‐of‐sight imaging, where scattering and structured illumination are intrinsic to the channel, spike‐coded event streams may provide an alternative to conventional intensity frames, and the same binary representation could be transmitted wirelessly with low error rates prior to decoding.

## Author Contributions

N.Z. and A.N. conceived the project. N.Z. and A.N. designed the experiments and methodology. N.Z. conducted the experiments and designed the computational toolkit. N.Z. and A.N. wrote the manuscript. N.Z. and A.N. contributed to the data analysis and provided feedback on the manuscript.

## Conflicts of Interest

The authors declare no conflicts of interest.

## Supporting information




**Supporting File 1**: advs77082‐sup‐0001‐SuppMat.docx.

**Supporting File 2**: advs77082‐sup‐0002‐SuppVideos.zip.

## Data Availability

The data that support the findings of this study are openly available in neuromorphic‐imaging at https://github.com/nzhang1218/neuromorphic‐imaging.
