# Supplementary material for: Neuromorphic Optical Tracking and Imaging of Randomly Moving Targets Through Dynamic Dense Scattering Media
Source: Adv Sci (Weinh). 2026 Aug 24:e77082. Online ahead of print. doi: 10.1002/advs.77082 (PMC13502347; doi:10.1002/advs.77082)
Supplement: Supplementary file 1 [file ADVS-9999-e77082-s002.docx]

**Supplementary**

**Table of Contents**

**Supplementary Notes**

**S1:** Preparation and Characterization of Turbid Media

**S2:** Results from Dynamic Fog Experiments

**S3:** Energy Consumption – Comparative Estimation between SNN and ANN-based Models

**S4:** Details of the SNN Model

**S5:** SNN Model - Ablation Studies

**S6:** Spiking Neuron: Leak-Integrate-and-Fire (LIF) Model

**S7:** Surrogate Gradient Function

**S8:** The DVS camera and Data Preprocessing

**S9:** Training and Evaluation of our SNN Model

**S10:** DVS Configuration and Synchronization

**S11:** SNN Training and Testing Environments

**S12:** State-of-the-art (SOTA) comparison

**S13:** Benchtop optical setup for experiments with solid phantom

**S14:** Temporal Bandwidth and Addressable Optical Thickness

**S15:** Field of View and Motion Sensitivity

**Supplementary Figures**

**Figure S1:** Experimental setup and characterization for dynamic fog imaging

**Figure S2:** Neuromorphic image reconstruction of a moving object unrecognizable to naked eye in dense fog

**Figure S3:** Summary of image reconstruction results obtained in transmission geometry (MNIST examples) in fog chamber

**Figure S4:** Benchtop optical layout for experiments deploying solid phantom as the turbid media

**Supplementary Tables**

**Table SI:** Comparing energy consumption between the SNN and ANN of the same overall architecture

**Table SII:** Feature ablation study on architectural components of ORM

**Table SIII:** Field of view and target-plane pixel pitch for the experimental configurations

**Table SIV:** Minimum resolvable target speed versus pixel pitch and SNN time-step

**Supplementary Videos (M1–M4)**

**Supplementary Video M1:** Tracking and reconstructing randomly moving MNIST characters via an event-driven neuromorphic optical system and deep SNN (transmission geometry).

**Supplementary Video M2:** Reconstructing stationary objects KMNIST via an event-driven neuromorphic optical system and deep SNN (reflection geometry).

**Supplementary Video M3:** Reconstructing randomly moving birds behind turbid water via an event-driven neuromorphic optical system and deep SNN (transmission geometry).

**Supplementary Video M4:** Fog chamber experiments showing dynamic drifting fog with variable optical thickness.

**Supplementary References**

**S1: Preparation and Characterization of Turbid Media**

The scattering strength of the turbid media used in this work was characterized using the optical thickness (OT) as the measured quantity, OT related to the number of photon mean free paths (MFP). Experimentally, the optical thickness at time t is defined from attenuation of the collimated transmitted optical power as

$$OT(t) = - log(P_{t}(t)/ P_{0})$$

where $P_{0}$and $P_{t}$ denote the power measured without scattering medium and detected optical powers at time t, respectively. Independently, the scattering mean free path is defined as $\mathcal{l}_{s}=1/\mu_{s}$, where $\mu_{s}$is the scattering coefficient. The number of mean free paths across a scattering phantom of physical thickness $L$is therefore calculated as

$$N_{\mathrm{MFP}}=\frac{L}{\mathcal{l}_{s}}=\mu_{s}L$$

For solid slab phantoms, we constructed silicone-based phantoms with microscale SiO_2_ particles as Mie scatterers, strictly following the methods and materials described in references^1,2^ (briefly described below). In the experiments, the scattering coefficient of the phantom was fixed at µ_s_ = 6 mm⁻¹ and anisotropy number as g=0.9 ^16^ so that for a slab thickness of 12 mm the resulting number of MFP = 72 in the transmission geometry, MFP = 144 in the double-pass reflection geometry. The primary substance for creating optical phantoms is SiliGlass, a transparent silicone rubber procured from MBFibreglass (PlatSil SiliGlass), which has two components: part A (base material) and part B (hardener). To achieve specific optical properties, a black silicone pigment known as Polycraft Black Silicone Pigment is used as an absorber (diluted in part A at a ratio of 2272:1), and silica microspheres (440345, Sigma-Aldrich) are employed for scattering. Phantom preparation involves mixing part A of SiliGlass with the diluted absorber for absorption and part B with the disaggregated microspheres for scattering. Both mixtures are stirred magnetically and ultrasonicated for 15 minutes each. After combining at a controlled temperature of 21°C, they are stirred for uniformity and poured into a 3D-printed mold.

For macroscale scattering media, we constructed two phantoms: (1) turbid water, generated by diluting 20% Intralipid (IL20) in a 30 cm^3^ size water tank; and (2) dynamic fog, implemented using a custom-designed 35 cm^3^ size fog chamber with separate fog generation and containment units. Fog was produced using a vaporizer with adjustable output and was introduced into and removed from the main chamber via two independently programmable fans. By controlling the fan speeds, the optical thickness within the chamber could be dynamically modulated.

**S2: Results from Dynamic Fog Experiments**

Figure S1: Experimental setup and characterization for dynamic fog imaging. (a) Photograph of the benchtop setup showing the fog chamber and cameras. FB: frame-based camera. OT Laser: to quantify OT in real time. (b) Example frames captured by the monochromatic frame-based camera under varying fog conditions, quantified by Optical Thickness (OT). (c) Real-time monitoring of fog OT. This demonstrates the dynamic nature of the scattering medium. Data was acquired only during the indicated "measurement time" window (light blue).

The benchtop experiments were conducted using a transmission geometry setup (Fig. S1, Fig. 2 of main text). Structured illumination, in the form of MNIST-derived digits moving along random trajectories in the transverse plane, was projected from an LCD screen through a 35 cm^3^ fog chamber. A Dynamic Vision Sensor (DVS) camera (Prophesee Gen 3.0 EVK) and a frame-based monochromatic camera (U3-30C0CP-M-GL, IDS) simultaneously imaged these moving targets from the opposite side of the chamber. Inherent polarization leakage through the LCD's liquid crystal polarizers introduced simulated (but real) background noise. Figure S1(b) presents sample frames from the frame-based camera at various OTs, illustrating that targets first become difficult then impossible to recognize around OT ≈ 4. The temporal variation of the fog's OT during the imaging session, shown in Fig. S1(c), confirms the dynamic nature of the scattering medium. Please refer to Supplemental video M4 for dynamic fog build-up illustration.

Fig. S2(a) illustrates the raw spikes recorded across DVS pixels as an x-y-t space-time graph. The raw spikes (positive and negative) were preprocessed into spiking tensors (upper part of panel (b) in the format *[T, C, H, W]*, where *T* labels the time steps, *C* is the number of channels, and *H, W* corresponds to the spatial dimensions. Object tracking by the optical tracking module (lower part of panel S2(b)) is shown as inferred object position (green crosses) when compared against the ground truth positions (red crosses). Panel 2(c) illustrates the reconstructed images (integrated outputs of the OTM and ORM modules) from time steps T1 to T18. During the initial time steps (T1 to T6), the reconstruction appears blurred and lacks discernible shapes. Such behavior is reminiscent of the human visual process where temporal memory plays a role in object recognition such as the brain building a recognizable image of a moving target in conditions of weak ambient light. Note that our SNN model leverages temporal memory, utilizing early-stage input data to progressively refine its understanding and recognition of the object. Across multiple time steps, the reconstruction process converges, with the object emerging at high resolution.

Figure S2. Neuromorphic image reconstruction of a moving object invisible to naked eye in dense fog. (a, b) Data and results for an MNIST digit '8' moving randomly within the scattering medium as an x,y,t plot. (a) Sample of raw spiking data from DVS pixels. (b) Spiking tensor derived from the preprocessed spikes, represented in the form [T, C, H, W] in upper panel. Object tracking, obtained from the OTM, illustrates the spiking neural network (SNN) inference (green crosses) alongside the ground truth positions (red crosses) of the randomly moving object in the lower panel. (c) Object reconstruction with tracking is the combined results from OTM and ORM at each time step. During a total 18 time steps the image of the object emerges as clear and fully recognizable.

Figure S3. Summary of fog chamber reconstruction results obtained in transmission geometry for randomly selected examples from MNIST, displayed as an 8×8 array where each image represents a distinct object. The figure compares the "ground truth" data, the raw spiking data recorded by the DVS camera through the fog chamber, and the final SNN-generated reconstruction. The input image array shows the temporally accumulated spike tensor across all time steps, reflecting the random motion trajectories and unrecognizable blurriness caused by the scattering media. The ground truth image array displays the spatial information of the objects, while the reconstructed image array shows the reconstructed target at the final time step. Quantitative evaluation yields an SSIM of 0.9340 and an MSE of 0.0178, demonstrating the efficacy of this approach. Note, the size of objects in ground truth and reconstruction image are magnified for visualization purposes.

Fig. S3 summarizes the reconstruction results obtained in transmission geometry for 64 randomly selected objects from the MNIST set, aggregated here as an 8×8 array where each image represents a distinct object which executed a trajectory of random motion for the same event duration (epoch). The first 10,000 characters from the full training set and 2,000 characters from the testing set collected from the optical experiments were used for training and testing, respectively. Using a standard computer equipped with an NVIDIA RTX 3090 GPU, training typically required approximately 6 minutes per epoch, while inference time averaged <15 ms for all time steps (e.g. 18 or 36 time steps representing 50–800 ms depending on the duration of each target) of a single target. The measured (SSIM) of 0.9340 and a mean squared error (MSE) of 0.0178 across the full testing dataset support the neuromorphic imaging strategy.

**S3: Energy Consumption – Comparative Estimation between SNN and ANN-based Models**

We computed the energy consumption of our SNN engine and compared it with a traditional ANN of the same overall architecture under the specific conditions of our experiments (Table SI). The computations are categorized into MAC (Multiply-Accumulate) operations, which involve floating-point data processing (including one multiplication and one addition per operation), and AC (Accumulate) operations, which consist solely of additions and are primarily used for processing binarized spiking data in the SNN.

In the SNN design, following the first spiking neuron layer, all subsequent data flows in the form of spikes (binary values: 0 or 1), resulting in AC-dominant operations and hence higher computational efficiency. In contrast, ANN architectures maintain floating-point data throughout the network, making MAC operations predominant. Furthermore, SNNs operate on an event-driven basis, where computations occur only when spikes are generated. This feature, combined with the sparse firing activity of spiking neurons, reduces both computational load and energy consumption.

In a comparative evaluation of our SNN design, we applied inference looping through all test data and monitored the average spiking rate, i.e. evaluated the ACs and MACs for every layer of the SNN as well as the ANN engines, respectively, using the same methodology described by Chen et al^3^. To facilitate a head-to-head comparison, LIF neurons in the SNN were replaced with ReLU neurons in the ANN while maintaining the same architecture. The total energy consumption was estimated using the equation below

$$E_{total}=T\times[{O_{MAC}\times E}_{MAC}+{O_{AC}\times E}_{AC}]$$

Where T represents the total number of time steps, $O_{MAC}$, $O_{AC}$ are the number of MAC and AC operations at each time step, respectively. $O_{AC}$ depends on the current spiking rate in each layer. We computed the estimated energy cost in 45 nm CMOS chip technology based on previous works^4–6^: the energy cost $E_{MAC}$, $E_{AC}$for each MAC and AC is 4.6 pJ and 0.9 pJ respectively^7^. Both SNN and ANN designs require multiple time steps for tracking and reconstruction, but the SNN demonstrates significantly lower energy consumption (7.99 mJ, nearly 18× lower than the ANN) due to its event-driven computation, sparse firing activity, and reliance on AC operations.

Table SI. Comparing energy consumption between the SNN and ANN of the same overall architecture, showing the dominance of AC operations in the SNN vs. MAC operations in the ANN, respectively. The last column highlights the energy benefits of the event-driven, sparse firing of our SNN model. Params: total parameters in the network. OPs: total operations. ACs and MACs are total number of MAC and AC operations over all time steps.

| **Model** | **Input Resolution** | **Params(M)** | **OPs(G)** | **ACs(G)** | **MACs(G)** | **Energy (mJ)** |
| --- | --- | --- | --- | --- | --- | --- |
| SNN | [18,2,64,64] | 3.69 | 34.81 | 4.95 | 0.769 | 7.99 |
| ANN | [18,2,64,64] | 3.69 | 30.94 | 0 | 30.94 | 142.32 |

**S4: Details of the SNN Model**

**Overview and Training Strategy**

**We developed a multi-module deep SNN architecture specifically designed for simultaneous tracking and image reconstruction of dynamic objects through scattering media.** The SNN processes asynchronous spike trains from the DVS camera to produce both object location coordinates and reconstructed images at discrete time steps. A particular challenge for SNNs is the non-differentiable nature of the various spiking neuron models which complicates the training process that use standard backpropagation techniques^8,9^. Common methods to train an SNN are: (1) Convert ANNs to SNNs^10^, (2) Train with Local learning rules^11^, and (3) deploy backpropagation with a surrogate gradient^8^ adapted to the spiking domain. In this work, we chose the surrogate gradient method to train our SNN model (see Method for implementation details) primarily as this allows end-to-end training that makes effective use of temporal information in the input.

A standard leaky integrate-and-fire (LIF) neuron model was chosen to incorporate time-dependent dynamics and discrete spike outputs, mimicking biological neurons more closely than neurons in ANNs (see S6). The LIF neurons maintain membrane potentials as internal states, providing inherent temporal memory crucial for integrating sparse event sequences from the DVS over multiple time steps. This statefulness fundamentally differs from ANNs, where neurons process inputs instantaneously without maintaining internal states.

**Network Architecture**

Our goal was to design an algorithm specifically capable of both tracking and imaging dynamic objects obscured by scattering media. The input data reflects changes in the scattered photon population registered by the DVS camera. These changes may be small and accompanied by significant background noise. After the very first hardware layer of denoising and feature extraction in the DVS, the subsequent software model should be able to locate objects, discover and extract key representations out of the background to achieve sharp image reconstruction. More generally, the SNN must be able to efficiently process both spatial and temporal features for practical event-driven imaging and be deployable on neuromorphic hardware. The network processes event sequences which were discretized into T time steps (for example, T = 18 for 360 ms trajectories for MNIST transmission experiment), where each step represents Δt_step = 20 ms of real-world dynamics. This temporal discretization is a current software necessity for gradient-based training.

In an SNN model (see Fig 3 in main text), the spiking information propagates as spikes and the output is reconstructed as tracked object images over a time series: $Y_{\text{out}}(T)=f_{\text{SNN}}(P(x,y,p,t);\text{ }\omega)$, where ω represents learnable parameters, $P\left( x,y,p,t \right)$ is a spike train (x, y, polarities, time), $P(\cdot)$ denotes the preprocessing pipeline that converts raw DVS events into spike tensors $X \in\mathbb{R}^(T\times2\times H\times W)$ for training and inference, and $Y_{\text{out}}\left( T \right)$ denotes reconstructed images at time-step $T$.

**SNN Object Tracking Module (OTM)**

The primary objective of tracking is to estimate the center coordinates of a randomly moving object at each time step. The Object Tracking Module [OTM] in lower portion of Fig. 3 takes the incoming spikes and outputs the predicted coordinates [x, y] in the 2D plane (this work focused on 2D motion; extending the approach to depth perception is a goal of future studies). The OTM framework consists of two main components: an encoding path and a linear mapping path. The encoding path incrementally extracts critical spatial information and encodes it into a latent representation, while the linear mapping path transforms the flattened latent data into the desired coordinate format [x, y] through a series of fully connected layers.

The encoding path uses a sequence of convolutional SNN blocks, each followed by a downsampling step. We denote one such single-convolution SNN block as:

$$\boldsymbol{SNN\_Block}=\boldsymbol{SN}\left( BN\left( Conv2d\left( x \right) \right) \right), [1]$$

where Conv2d(⋅) is a 2D convolution, BN(⋅) denotes batch normalization, and SN(⋅) is a spiking neuron operation **(here LIF neurons)**. Starting from the input $x_{0}^{*}=x$, each **encoded** output $x_{i}^{*}$ is obtained via:

$$x_{i}^{*}=\bar{\boldsymbol{DS}}\left( \boldsymbol{SNN\_Block}\left( x_{i-1}^{*} \right) \right),x_{0}^{*}=x; x_{i}^{*}\in\mathbb{R}^{T\times C_{i}\times\frac{H}{2^{i}}\times\frac{W}{2^{i}}}; i=1, \ldots, n [2]$$

**Here**, $\bar{DS}$ is a **downsampling** operator (e.g., maxpooling) that halves the spatial dimensions (H, W) at each stage, and T is the number of time steps **processed by** the network.

**Once** the encoding path **has** generated a latent representation, **the** linear mapping **path** converts the **flattened** features into [x, y] coordinates. Each fully-connected (FC) SNN layer is given by:

$${x_{j}^{*}}=\boldsymbol{SN}\left( FC\left( x_{j-1}^{*} \right) \right), x_{j}^{*}\in\mathbb{R}^{T\times C_{j}} , j=1, \ldots, m [3]$$

where FC(⋅) is a fully connected layer. After the last block, the final output layer **provides** two neurons corresponding to the [x, y] coordinates normalized to the range [0,1].

The output is derived from the membrane potential of the last spiking neuron (SN) layer. At each time step T_i_, the last SN layer accumulates spikes from the preceding layers to update its membrane potential. Notably, only the last SN layer undergoes resetting at each time step while the rest of the network remains unreset. This resetting strategy is employed for two primary reasons: (1) Handling random object movement: The tracked object may move unpredictably, sometimes transitioning from a position with a high normalized value to one with a low normalized value. While the SN layer effectively accumulates incoming spikes to increase its membrane potential, it is less efficient at rapidly decreasing the potential as needed. Although a constant potential decay occurs due to the leakage mechanism, this decay may be insufficient for substantial reductions in membrane potential. Without resetting the last SN layer, such scenarios could result in incorrect output estimations. (2) Preserving memory in intermediate layers: The remaining layers in the network do not require resetting because the network is trained over a series of time steps. The “stateful” parameters, such as the membrane potentials of intermediate SN layers, act as memory elements in the LIF model. These parameters capture contextual information from both previous and current positions, enabling the network to leverage temporal dependencies and achieve higher accuracy in predictions. The OTM's lightweight architecture enables rapid and accurate tracking at each time step while reconstruction requires temporal integration. This asymmetry reflects the fundamental difference in task complexity: localizing the center of event activity requires less information than reconstructing detailed object structure.

**SNN Object Reconstruction**

The SNN Reconstruction Module includes two sub-modules for final object reconstruction (upper portions of Fig. 3 in main text). The first, an Object Reconstruction Module (ORM) follows a U-Net-like ^12^ encoder-decoder structure which provides coarse spatiotemporal reconstruction. Inspired by BASNet design ^13^, a multiscale Residual Refinement Module (RRM) follows after the ORM to refine the resulting reconstruction by learning the residuals between the ORM reconstruction and the groundtruth (ablation studies in Results section).

**Object Reconstruction Module (ORM)**

An encoder-decoder structure is chosen because it can computational efficiently capture both global context and local details while discarding noise and irrelevant details^12,13^. ORM consists an encoding path progressively downsampling the spatial dimensions of the input feature maps and a decoding path reconstructing the output at the original resolution, incorporating skip connections to preserve fine-grained spatial details from early stages. At the center of the network, where the downsampling reaches the deepest level, i.e. a bottleneck where the spatial dimensions are smallest, the model captures the most important features of the input spikes.

Mathematically, the ORM can be expressed as:

***For the i-th encoding blocks (Residual SNN Block with Downsampling):***

$$x_{i}^{*}=\bar{\boldsymbol{DS}}[\boldsymbol{SN}\left( BN\left( Conv2d\left( \boldsymbol{SNN\_Block}\left( x_{i-1}^{*} \right) \right) \right)+ \boldsymbol{SNN\_Block}\left( x_{i-1}^{*} \right) \right)],$$

$x_{0}^{*}=x; x_{i}^{*}\in\mathbb{R}^{{T\times C}_{i}\times\frac{H}{2^{i}}\times\frac{W}{2^{i}}};i=1,\ldots, n. [4]$

***For the j-th decoding blocks (Double-Conv SNN Block with Upsampling):***

$y_{j}^{*}=\boldsymbol{SNN\_Block(SNN\_Block(}\bar{\boldsymbol{US}}\left( y_{j-1}^{*} \right)+\boldsymbol{SKIP))}, \left[ 5 \right]$

$${SKIP=x_{n-j}^{*}; y}_{0}^{*}=x_{n}^{*};y_{j}^{*}\in\mathbb{R}^{T\times C_{j+n}\times\frac{H}{2^{n-j}}\times\frac{W}{2^{n-j}}}; j=1,\ldots,n.$$

***Output block:***

$$Output=\boldsymbol{SN}(Conv2d(y_{n}^{*})), Output\in\mathbb{R}^{T\times1\times H\times W}; y_{n}^{*}\in\mathbb{R}^{T\times C_{2n}\times H\times W}$$

where $I\in\mathbb{R}^{T*C\times H\times W}$is the input data after preprocessing, and *T*, $C, H, W$ denote the timestep, channels, height, and width, respectively. SKIP represents skip connection from the encoding blocks, concatenating the up-sampled spikes from the decoding path.

The encoding path consists of multiple *encoding blocks*. Each *encoding block* includes a residual SNN block and a downsampling (DS) layer, which progressively reduce the spatial resolution while increasing the number of feature channels. Each residual SNN block contains double-convolution layers, batch normalization (BN) layers, and spiking neuron layers $\mathrm{SN}\left( \cdot\right)$.

In the decoding path, the network employs multiple *decoding blocks* which consist of upsampling (US) layers to gradually restore the spatial resolution. The US layers are interleaved with double-conv based SNN blocks to converge the reconstructed feature maps. Skip connections are utilized between corresponding layers in the encoder and decoder to fuse high-resolution features from the encoding path with the upsampled features in the decoding path.

In our method, the US layer is a bilinear layer or a transposed convolution layer. The DS layer is a maxpooling (MP) layer. The DS/US layer performs spatial reduction/restoration by a factor of 2.

The final output block includes a convolutional layer followed by a final SN layer. The convolutional layer transforms channels into 1 (gray image). This last SN layer processes the reconstructed spike events into the final output (membrane voltage or spikes) for training and inference.

A stateful synapse filter is added after each residual SNN block and double-conv SNN block. By placing it to filter the output current, the network can acquire stateful synapses which further enhance its memory ability^14^.

We emphasize how spiking neuron layers $\mathrm{SN}\left( \cdot\right)$ serve as a crucial and unique component in our architecture, efficiently encoding spatiotemporal information into membrane potentials and subsequently transforming them into binary spikes. This process enables spike-driven computation in subsequent layers, aligning with the event-based nature of our input data. Each $\mathrm{SN}$ layer, built from LIF neurons, takes spikes from the previous layer, updates its membrane potential (tensor U), and fires spikes (tensor S) as output: $S = SN(U)$.

**Residual Refinement Module (RRM)**

The reconstruction output from the ORM is a probability map, where each pixel value ranges between [0, 1], representing the likelihood of reconstruction at that location. However, the probability predictions from ORM are prone to two common issues: (1) blurry and noisy boundaries, and (2) uneven regional probabilities^13^.

To mitigate these problems, a Residual Refinement Module (RRM) is incorporated following ORM. The decision to split reconstruction into two consecutive modules—rather than asking a single network to produce the final map directly—follows from the physics of imaging through dense scattering media. The photons that carry target information are those that interacted with the target before continuing through the medium; their number is intrinsically limited, and each undergoes tens to hundreds of further scattering events before detection. The target therefore appears at the sensor as a small optical perturbation on a much larger background of multiply-scattered photons. The coarse silhouette survives this process because relatively many target-interacting photons contribute to it, whereas fine spatial features (the thin strokes of a Kanji character, the slender limbs of a bird) involve far fewer such photons and arrive with much lower signal-to-noise. Recovering the target is thus an ill-posed inverse problem that decomposes naturally into two stages: first recover the coarse layout from the higher-SNR component of the signal (the ORM), then restore the finer detail that scattering and noise have suppressed (the RRM). Decoupling the two stages lets each branch be supervised independently at the SUP nodes and specialize on a single objective, rather than balancing coarse-silhouette recovery and fine-residual learning within one set of weights. Because the RRM operates only on a low-dimensional residual, it can be made shallow and inexpensive—its parameter count and AC operations are a small fraction of the ORM’s—preserving the energy advantage of the SNN reported in Table SI. The quantitative benefit of this split is documented by the ablation study in S5 (Table SII).

The structure of the RRM is simpler than that of the ORM, consisting of an input block, three encoder blocks, a bridge, three decoder blocks, and an output block. The RRM is designed to learn the residual differences between the ORM's output and the ground truth, following the formula:

$R_{final}=R_{coarse}+R_{residual}$	[6]

Where $R_{coarse}$ is coarse reconstruction generated by the ORM; $R_{residual}$ is output from the RRM prior to adding the input; $R_{final}$ is the final refined reconstruction produced by the model.

Our image reconstruction model demonstrates two key unique features: (1) Memory Capability:
The model is built on a SNN instead of an ANN. Unlike ANN neurons, spiking neurons such as LIF neurons maintain internal states that store memory across time steps. This memory capability is further enhanced by the use of synapse filters, which transform synapses into stateful synapses. Consequently, both neurons and synapses in the model contribute to its memory retention. (2) Training Over Multiple Time Steps: The network is trained across multiple time steps rather than just a single time step, enabling it to handle the random spatial and temporal motion of obscured objects. Since a randomly moving object may appear in different locations at each time step, the model requires robust memory to contextualize the detected spiking patterns over time. This is crucial for addressing motion variance in DVS detection, where the same object can generate entirely different spiking patterns due to variations in its movement direction, acceleration, and other motion-related factors. Furthermore, different objects may exhibit similar spiking patterns under varying conditions. Thus the network's ability to retain memory of spiking patterns across time ensures it can learn and reconstruct objects effectively.

**Hybrid Loss Functions for Training**

In the SNN Reconstruction Module, the loss calculation is made at multiple stages (denoted as "SUP" in Fig. 3 of main text). At each SUP stage, the membrane potential from the last spiking layer is upscaled to match the input's shape and compared with the ground truth for loss calculation. This multi-stage loss strategy is implemented to mitigate overfitting and ensure robust learning^15^. A total of five loss calculations are performed at various stages, including outputs from both the ORM and RRM modules. To achieve accurate and high-quality reconstructions, globally and pixel-wise, a hybrid loss function is employed, comprising three components:

$$\mathcal{L}_{\text{recon}}=\lambda_{\text{BCE}}\mathcal{L}_{\text{BCE}}+\lambda_{\text{SSIM}}\mathcal{L}_{\text{SSIM}}+\lambda_{\text{IoU}}\mathcal{L}_{\text{IoU}}$$

(a) Binary Cross-Entropy (BCE) Loss^16^ evaluates pixel-level accuracy by comparing each pixel in the reconstruction with the corresponding ground truth value.

$$\mathcal{L}_{\text{BCE}}=-\frac{1}{HW}\sum_{i,j} [y_{ij}\log(\hat{y}_{ij})+(1-y_{ij})\log(1-\hat{y}_{ij})]$$

(b) Structural Similarity (SSIM) Loss^17^ assesses reconstruction quality at the patch level, ensuring structural coherence and perceptual similarity to the ground truth.

$$\mathcal{L}_{\text{SSIM}}=1-SSIM(\hat{y},y)$$

(c) Intersection over Union (IoU) Loss^18^ measures overlap at the map level, encouraging better overall reconstruction coverage and accuracy.

$$\mathcal{L}_{\text{IoU}}=1-\frac{\sum_{i,j} \hat{y}_{ij}\text{ }y_{ij}}{\sum_{i,j} (\hat{y}_{ij}+y_{ij}-\hat{y}_{ij}y_{ij})}$$

Where $\boldsymbol{y}_{\boldsymbol{ij}}$ is groundtruth value and ${\hat{\boldsymbol{y}}}_{\boldsymbol{ij}}$is predicted value. We empirically set $\boldsymbol{\lambda\_BCE = \lambda\_SSIM = \lambda\_IoU = 1}$ after initial tuning, finding this equal weighting provided robust performance across datasets without requiring dataset-specific hyperparameter optimization.

Within the SNN Tracking Module, the loss calculation involves comparing the predicted center coordinates with the ground truth center coordinates. The Mean Square Error (MSE) is used as the loss function to minimize the error between predicted and true center positions, facilitating precise tracking (details in Methods). We evaluated L1 norm (Manhattan distance) as an alternative but found L2 norm (MSE) provided superior convergence:

$$\mathcal{L}_{\text{track}}=\left\| [ \hat{x} , \hat{y} ] - [ x_{\text{gt}} , y_{\text{gt}} ] \right\|_{2}^{2}$$

The tracking and reconstruction modules were trained separately with its loss. During inference, both modules process the same input tensor in parallel, with the combined output generated once both complete their computation. The overall inference latency is determined by the slower reconstruction pathway (<15 ms for T=18 steps on RTX 3090 GPU).

**S5: SNN Model - Ablation Studies**

Across all ablation experiments, we systematically perturbed one design axis at a time to understand which ingredients are most critical for reconstructing targets through scattering media. Starting from a baseline ORM U-Net–style SNN, we explored 21 configurations spanning architectural components, loss formulations, neuron time constants, and synapse filters, all trained under identical conditions and evaluated with SSIM, PSNR, MAE, and MSE. The architectural study shows a clear hierarchy of impact: upgrading the encoder to a ResNet backbone provides the single largest boost in quality, while adding the Residual Refinement Module (RRM) further sharpens object boundaries and cleans up coarse artifacts. Synapse filters with a fixed time constant (τ = 2.0) give another consistent gain, indicating that shallow temporal smoothing at the feature level is beneficial. In contrast, multi-scale supervision on its own slightly hurts performance, likely because additional auxiliary heads introduce gradient interference unless paired with other stabilizing components. Quantitatively, relative to the ORM-only baseline, adding the RRM yields a consistent improvement of +0.1% in SSIM, +0.69% in PSNR, +2.44% in MAE and +2.71% in MSE (Table SII); visually it produces a sharper target outline and removes the regions of intermediate probability that the ORM otherwise leaves around the target.

On the optimization side, the loss ablations highlight that *what* we ask the network to optimize can be as important as *how* it is built. A BCE+IoU combination emerges as the most reliable objective: removing IoU leads to catastrophic collapse of spatial structure, confirming that explicit overlap-based supervision is indispensable for recovering thin edges and silhouettes behind fog. Perhaps more surprisingly, including SSIM as part of the training loss actually degrades all downstream metrics, even the final SSIM itself, suggesting that SSIM’s gradients conflict with BCE/IoU in this regime and push the network toward suboptimal local minima. BCE alone can drive a relatively high PSNR but at the cost of perceptual and structural fidelity, reinforcing that a multi-objective loss is necessary to maintain both pixel-level accuracy and shape consistency.

Finally, the temporal ablations clarify how much memory the SNN should have when integrating DVS streams. Relative to an integrate-and-fire baseline, leaky integrate-and-fire neurons with a moderate membrane time constant (τ = 1.0) yield the best trade-off between temporal smoothing and responsiveness; longer integration (τ = 10.0) over-smooths dynamics and actually underperforms the IF case. A similar conclusion holds for synapse filters: a fixed τ = 2.0 outperforms both other fixed values and fully learnable time constants, implying that a simple, domain-informed temporal prior is more effective than giving the optimizer too much freedom. Together, these results led to our final configuration: a ResNet-encoded ORM with RRM, fixed synapse filtering, LIF neurons with τ ≈ 1.0, and a BCE+IoU loss—an architecture that is both performant and conceptually well-grounded for event-based reconstruction through scattering media.

Table SII. Feature ablation study on architectural components of the Object Reconstruction Module (ORM). Quantitative comparison of ORM variants were evaluated on the fog-chamber image reconstruction task. Starting from the baseline U-Net–style SNN encoder–decoder, we incrementally add (i) the Residual Refinement Module (RRM), (ii) a ResNet-based encoder, (iii) synapse filters with fixed time constant τ = 2.0, and (iv) multi-scale upsampling and supervision. For each configuration, the table reports SSIM, PSNR, MAE, and MSE, highlighting how residual refinement, stronger encoder features, and temporal synapse filtering contribute to reconstruction quality, while multi-stage supervision on its own can slightly degrade performance.

**S6: Spiking Neuron: Leak-Integrate-and-Fire (LIF) Model**

LIF is selected to be the basic neuron model of our deep SNN. It incorporates time-dependent dynamics and discrete spike outputs, mimicking biological neurons more closely comparing to neurons in common ANNs. An LIF maintains an internal state (membrane potential), exhibits a leakage of potential over time, fires spikes when a threshold is reached, followed by a refractory period. The LIF neuron dynamics are well-known:

$$V\left[ t+1 \right]=V\left[ t \right]+\frac{\Delta t}{\tau_{m}}\left( -\left( V\left[ t \right]-\mathrm{Vrest} \right)+\sum_{i} w_{i}x_{i}\left[ t \right] \right) \left[ 8 \right]$$

Where V(t) is the membrane potential at time t; $\tau_{m}$is the membrane time constant, representing how quickly the neuron’s membrane potential decays towards the resting potential in the absence of input; Vrest is the resting membrane potential; $w_{i}$ is the synaptic weight from presynaptic neuron I; $x_{i}$[t] is the spike train of presynaptic neuron i at time t; Δt is the time step used for numerical integration. This is a discrete-time modeling approximation for computer simulations. The equation [8] can be written as:

$V\left[ t+1 \right]=\beta V\left[ t \right]+\sum_{i} \bar{w}_{i}x_{i}\left[ t \right] \left[ 9 \right]$

Where $\beta=1- \frac{\Delta t}{\tau_{m}}$ , represents the decay parameter; $\bar{w}_{i}= \frac{\Delta t}{\tau_{m}} w_{i}$ is the weight with decay. When membrane potential $V\left[ t \right]$ crosses a threshold $V_{th}$, the neuron generates a spike, and the membrane potential is reset to resting potential.

$$S\left[ t \right]=\Phi\left( V\left[ t \right]-V_{th} \right) \Phi\left( x \right)=\left\{ \begin{aligned} 1, x\geq0 \\ 0, x<0 \end{aligned} \right. \left[ 10 \right]$$

In contrast, ANN neurons, such as those using the ReLU, Sigmoid or Tanh activation functions, are simplified models, process inputs instantaneously without maintaining an internal state, producing continuous output values instead of discrete spikes without a refractory period. While the simplicity and efficiency of ANN neurons have made them the standard in current deep learning ANN architectures. The biological realism of LIF and other SNN neurons makes them more valuable for neuromorphic computation, gaining advantages in sparsity, energy-efficiency and handling complex neuronal simulations.

**S7: Surrogate Gradient Function**

Gradient needs to be calculated when train a SNN using gradient descent. However, the derivative of the LIF neuron’s Heaviside function $\Phi\left( x \right)$ (equation [10],[11]) returns either +∞ when x = 0, or 0 when x ≠ 0, which prevents learning. This issue is commonly referred to as the “dead neuron problem”. To train our SNN network, a surrogate function $\sigma(x)=\frac{1}{\pi}arctan(\frac{\pi}{2}\alpha x)+\frac{1}{2}$ [arctangent (ATan)] is used to replace the Heaviside function $\Phi\left( x \right)$. ATan function is continuous and differentiable, making it suitable for backpropagation in SNNs. This approach helps to bridge the gap between the biological plausibility of SNNs and the effectiveness of traditional learning methods, allowing SNNs to benefit from gradient-based optimization.

**S8: The DVS camera and Data Preprocessing**

Instead of capturing static frames, a DVS records changes (events) in incident intensity at each pixel independently and asynchronously, operating on the logarithm of intensity. An event of polarity $p$is emitted at pixel $\left( x , y \right)$once the log-intensity change since that pixel's previous event crosses a contrast threshold $C$:

$$p\left( x,y,t \right)=\left\{ \begin{matrix} +1 & \Delta L\left( x,y,t \right)\geq+C \\ -1 & \Delta L\left( x,y,t \right)\leq-C \\ 0 & \text{otherwise,} \end{matrix} \right. \Delta L\left( x,y,t \right)=L\left( x,y,t \right)-L\left( x,y,t-\Delta t \right) [11]$$

where $L(x,y,t)=logI(x,y,t)$ is the log-intensity, $t-\Delta t$ is the time of the previous event at (x, y), and $C>0$ is the contrast threshold setting the sensor's sensitivity. Expressed as an equivalent fractional contrast $\theta_{C}=e^{C}-1$.

The value of $\theta_{C}$ sets the operating point of the entire pipeline, because every spike that reaches the SNN must survive both the scattering medium and the threshold gate. Lowering $\theta_{C}$ recovers more of the small optical-intensity changes that survive scattering, strengthening the aggregate signal that drives both tracking and reconstruction, but at the cost of increased shot noise, more background events, and a finite risk of saturating the event stream where the local intensity change is most intense. Conversely, raising $\theta_{C}$ suppresses noise events but also rejects the low-contrast perturbations produced by targets that the medium has already attenuated: only those pixels whose temporal contrast exceeds the threshold fire, so the SNN receives an underpopulated spike tensor — visible, for example, as missing thin strokes in the reflection-geometry Kanji reconstructions of Fig. 4. In the double-pass reflection geometry (effective OT ≈ 6.6, ≈ 144 MFPs) and in dense fog (OT ≈ 4), multiple scattering leaves only a small optical perturbation on top of a large background of incoherently scattered photons, so sensitivity rather than noise rejection becomes the binding constraint. We therefore biased the DVS to its lowest stable setting, $\theta_{C}$ ≈ 12% (default 25%; see S10). Because the accessible range is bounded from below at ≈ 12% by the commercial sensor, a systematic sweep of $\theta_{C}$ toward smaller values—where the most informative regime for low-contrast targets would lie—is not currently possible; we expect this to become accessible with emerging high-sensitivity event sensors (see Discussion).

The generated binary spike events along the temporal axis can be organized into a stream consisting of $N$ spike events, expressed as a tuple: $\left\{ e_{i} \right\}_{i=1}^{N}=\left\{ \left( x_{i},y_{i},t_{i},p_{i} \right) \right\}$, where p signifies spike polarity (+1 or -1; increasing or decreasing light intensity), x and y are coordinate values of that pixel in sensor’s x-y plane, t is time step in microsecond.

The inputs to our SNN model are spike input tensors preprocessed from the raw spike trains. We begin by selecting data region of interest (ROI) to only select spike trains from a certain number of sensor pixels. This is followed by applying a selection of noise filters: activity noise filter, spatial-temporal contrast filter, and antiflicker filter to remove unwanted spikes to retain only informative events caused by dynamical variations in the optical scene. Subsequently, the denoised spikes undergo a dimensionality reduction process; we chose here a bio-inspired insect eye method^19^ in reducing spatial dimension while preserving essential information. Lastly, the refined spike events are organized through a 3D binning process in both spatial and temporal domains, grouping them as a sequence of event time windows. An input tensor takes the form of $[T, 2, H, W]$ where T is the total number of time steps; channel 2 means both positive and negative polarities.

**S9: Training and Evaluation of our SNN Model**

As model targets in the benchtop optical experiments we chose two widely used datasets in computer vision: MNIST (hand-written digits) and K-MNIST (hand-written kanji characters). Each have geometrical features from which more complex objects can be composed. Both sets have 60,000 training and 10,000 testing data. We choose first 10,000 data from its original training dataset, and first 2,000 data from original testing dataset, as our training and testing datasets. This was a pragmatic choice: the reduced size of data can speed up benchtop experiments and computation process, while still sufficiently large to train the model.

The reconstructed image $R [T, 1, H, W]$ is obtained from the accumulated membrane potential from the spiking neuron layer of output block, i.e. the last time step in a series of time steps. The corresponding target images are from original datasets after preprocessing (resizing, binarizing, etc), to match the reconstructed image. Target images represent the full features of dynamic objects. In the experiments, a target image $G [T, 1, H, W]$ is a gray image at each time step. The detailed parameters employed in our model are listed in the table at right bottom shown in Figure 3. We refined our model using the *AdamW* optimizer over 50 epochs on the training dataset. Additionally, we conducted 5 iterations of training and testing, each with a different initialization. We confirmed that our models were adequately trained by analyzing their training and testing loss profiles. We observed no signs of overfitting as the testing loss consistently decreased alongside the training loss with each iteration.

In our implementation, the GPU processes all time steps in a sequence, with a total inference time on the order of 10 ms for one complete dynamic episode - substantially shorter than the physical duration of the benchtop events. The overall latency is therefore determined primarily by how long the DVS must observe the scene to accumulate sufficient informative events from the scattering medium for reconstruction, rather than by SNN processing time. For example, in MNIST transmission experiments (main text Fig 2b, 5), the DVS temporal resolution (at microsecond) is well in excess of the 100 Hz DMD pattern rate, and the SNN computation comfortably keeps pace with the event stream.

S10: DVS Configuration and Synchronization

We utilized the Prophesee Evaluation Kit 3.0 – VGA as our DVS camera. This camera has a sensor resolution of 640 x 480, with each pixel measuring 15 x 15 μm. It has a latency of 220 μs and a dynamic range exceeding 120 dB. By default, the nominal contrast threshold for detecting a new event is set to 25%. However, adjusting the sensor's bias can reduce this threshold to 12%^20^. To enhance photon collection, a C/CS mount lens system is mounted above the camera sensor. During our experiments, the lens aperture was maintained at f/2.8 to optimize light entry to the sensor.

We emphasize that $\theta_{C}$ is a nominal, derived quantity rather than a parameter that can be set directly on the sensor. On the Prophesee Gen 3.0 EVK it is governed by a combination of pixel-level bias settings (bias_diff_on, bias_diff_off, bias_diff, bias_fo, bias_hpf and bias_refr), so the effective contrast threshold for any given bias combination must be calibrated experimentally rather than read back from the device. The values quoted above—≈ 25% at the default biases, reduced to ≈ 12% at our lowest stable setting—were obtained in this way; the manufacturer’s bias-tuning documentation and community guidance both confirm that the per-bias threshold is not exposed as a single readable register. This is why we report $\theta_{C}$ as an empirically calibrated operating point rather than a directly programmed value.

The DVS camera is equipped with synchronization input and output capabilities, enabling the detection of external signals for coordinating between multiple devices. Here, a rising signal edge is interpreted as a start signal, while a falling edge signifies a stop signal. Throughout our experiments, the DVS camera remained operational, ensuring uninterrupted event capture. As part of our display procedure for a target, both the e-ink display and DMD (based on the experimental type) sent a 5V TTL square wave signal. This signal's rising edge preceded the display onset, and its falling edge marked the display conclusion. The DVS system recognized and timestamped these synchronization signals alongside events, offering a microsecond resolution. Once all targets were displayed, the accumulated spike trains (events) were retrieved and partitioned into distinct recordings, according to the identified trigger signals. Subsequently, each recording underwent preprocessing to filter out noise events and were tagged with the corresponding target identity, readying them for the subsequent training and testing phases.

S11: SNN Training and Testing Environments

We set up our training and testing environment with spikingJelly library^21^ and Lava framework^22^ for neuromorphic computing.

**S12: State-of-the-art (SOTA) comparison**

To position our work, we compare it against several state-of-the-art techniques. A SOTA comparison **table comparing different works is summarized here:** [**Link**](https://www.dropbox.com/scl/fi/2ijhau67ww6coafbm2wot/SOTA-table.png?rlkey=jtyj0mwhzfzydxpj1fejmcarq&dl=0) **to table**

**Speckle Correlation Techniques:** This family of methods exploits the statistical correlations within speckle patterns generated by light scattering, typically captured by standard intensity-based cameras.

**Shi et al., 2023** ^23^  utilizes *overlapping incoherent speckle correlations* arising from object motion for high-resolution (demonstrated 1µm) reconstruction and tracking. It operates with incoherent light but requires specific object motion relative to the speckle size and performs processing post-capture. Targets included resolution charts and fluorescent beads undergoing controlled motion.

**Jauregui-Sánchez et al., 2022** ^24^ focuses specifically on *real-time tracking* by subtracting speckle correlations between frames. This computationally efficient method works beyond the traditional memory effect range but does not inherently perform reconstruction. It used simple shapes (dots, triangles) on a DMD undergoing controlled motion.

**Shi et al., 2022** ^25^ adapts speckle correlation (RSESIC) for reconstructing moving objects (slits, numbers) obscured by complex, multi-corner scattering geometries ('random corridors'), demonstrating robustness in specific challenging static scattering scenarios.

**Xu et al., 2020** ^26^ investigates the feasibility of using *quantized (low bit-depth)* speckles for autocorrelation-based reconstruction and tracking of simple objects (letter 'X') undergoing controlled motion, highlighting potential for data compression and reduced hardware demands.

**Contrast*:*** Our method differs fundamentally by using a DVS, capturing sparse temporal events instead of intensity frames, and employing SNNs for processing, bypassing the need for direct correlation calculations. We demonstrated capability with naturalistic *random object motion*, a scenario less explored by these controlled-motion speckle methods.

**Deep Learning (ANN-based) Methods:** These approaches employ various Artificial Neural Network architectures (CNNs, RNNs, U-Nets, Transformers) typically processing data from standard cameras.

**Lyu et al., 2019** ^27^ used an HNN (CNN+DNN hybrid) for reconstruction through thick *static* scattering media, learning a mapping from speckle patterns to MNIST digits displayed on an SLM. It requires large datasets and was demonstrated for static targets.

**Ma et al., 2023** ^28^ employs a U-Net DNN processing the *difference* between consecutive speckle frames for simultaneous imaging and tracking of various objects (digits, letters, faces) undergoing controlled motion through static media.

**Liu et al., 2024** ^29^ utilizes a DNN (DescatterNet) for high-speed reconstruction of complex real-world objects (toys, outdoor scenes) through *dynamic* scattering media using incoherent light. It requires very large datasets and significant computational power (GPU) for its real-time inference but demonstrates strong generalization.

**Yan et al., 2024** ^30^ proposes a TP-RNN fusing temporal dynamics with phase spectrum information extracted from speckle sequences for imaging and trajectory *prediction* of MNIST digits undergoing controlled (uniform, accelerated, sinusoidal) motion through static media.

**Feng et al., 2023** ^31^ uses ANNs for guidestar-free wavefront shaping and reconstruction. It actively modulates the wavefront with an SLM to correct aberrations in static/dynamic media for various targets (USAF, stamps, cells).

**Contrast*:*** While powerful, these ANN methods process dense frame data and require substantial computational resources (often GPU-accelerated) and large training datasets (Lyu, Liu, Ma, Yan). Our DVS+SNN approach processes sparse event data, offering potential for significantly lower power and latency, especially on neuromorphic hardware. Our SNN's stateful neurons provide inherent temporal memory, contrasting with the explicit recurrent connections in Yan et al. or the frame-difference approach of Ma et al. We handle *random motion*, unlike the controlled trajectories in Yan et al. or Ma et al., and operate passively, unlike the active modulation in Feng et al.

**Specialized Optical Systems:**

**Kanaev et al., 2018** ^32^ combines nanosecond time-gating with coherent holographic detection (ICCD) and multi-pulse processing for reconstruction through extremely attenuating *dynamic* media (turbid water, fog). It relies on detecting scarce ballistic/near-forward photons from static targets ('X', 'THORLABS' plate) using pulsed coherent illumination.

**Contrast:** Our system uses simpler, continuous-wave incoherent illumination and detects scattered light using a DVS+SNN, offering a different operational regime focused on dynamic objects rather than maximizing penetration depth via ballistic photon selection.

**S13 Benchtop Optical Setup for Experiments with Solid Phantom**

Figure S4. Benchtop optical layout for experiments deploying solid phantom as the turbid media. (a) The beam from a low power infrared laser (λ= 850 nm) is first expanded, then divided by a 50-50 beamsplitter for experiments in reflection and transmission geometry, respectively. The “upper” beam in the figure applies to reflection experiments where a silicone-based scattering phantom with number of photon MFP = 72 is positioned in front of an E-ink display. The display is split-screen programmed to present a pair of duplicate targets (400 ms “flash”), one being obscured by the phantom and the other without any impediment to free space propagation. A DVS camera captures reflected photons from both targets simultaneously. In the transmission experiments, the “lower beam” illuminates a Digital Micromirror Device (DMD) which projects programmed sets of images onto the scattering phantom (MFP = 72) with the DVS camera now behind the phantom to capture transmitted photons. (b) Illustration of one Kanji character duplicate pattern generated by the E-ink display (left image), and room-light view of the reflected light with and without a phantom (right image). (c) A photo from the laboratory benchtop showing the positioning of the DVS camera and a phantom (MFP = 72) in transmission experiments. (d) Example of an image of the DMD projection of one Kanji character.

**S14: Temporal Bandwidth and Addressable Optical Thickness**

Here we summarize the upper temporal frequency and the optical thickness (OT) that the method can address. The fan-controlled fog dynamics of Fig. S1 are slow: the OT(t) trace is dominated by spectral content below ≈ 0.002 Hz (90% of the spectral power lies below ≈ 0.0023 Hz and 95% below ≈ 0.013 Hz), with the 3.5–4.5 OT swings of Fig. S1(c) evolving over a timescale of ≈ 2000 s. This drift is far below the temporal capability of the imaging pipeline.

The temporal capability is set by three independent factors. (i) The DVS timestamps individual events at microsecond resolution with a measured per-pixel latency of ≈ 220 µs (S10), supporting effective intra-scene event rates ≥ 10 kHz. (ii) The SNN processing time-step Δt = 20 ms used here places a Nyquist limit at ≈ 25 Hz; Δt is purely a preprocessing choice, and reducing it to 1–2 ms—which only increases the number of time steps N while leaving the architecture unchanged—extends the addressable medium-fluctuation bandwidth to ≈ 250–500 Hz. (iii) The total sequence duration *T*_duration_ = N·Δt (here 360 ms) sets the slowest resolvable fluctuation at ≈ 1/ *T*_duration_ ≈ 3 Hz, consistent with the smooth OT drift we observe.

There is a trade-off in choosing Δt. For a fixed *T*_duration_, reducing Δt improves temporal resolution but increases N proportionally, lengthening both training and inference; a smaller Δt also reduces the number of signal events per time step, which makes the model harder to train. An optimal Δt therefore resolves the target dynamics while preserving the efficiency of the pipeline. Within these bounds the system is matched to atmospheric, underwater, and biological scattering processes whose dominant fluctuations lie in the millihertz-to-kilohertz range.

The largest OT we demonstrate is the reflection-mode silicone-phantom configuration: each photon traverses the slab twice, so a single-pass OT of 3.3 corresponds to an effective total OT of ≈ 6.6 (≈ 144 MFPs) seen by an event-generating photon. At that OT the unprocessed scene is unrecognizable to the naked eye and to a conventional CMOS camera (main-text Fig. 4; Fig. S4), yet the SNN reconstructs the targets at SSIM = 0.81. Beyond this point the addressable OT is set by the budget of target-interacting photons that survive transport and reach the DVS, which falls approximately as $exp(-OT)$. Further extension is largely a hardware question—raising illumination power, coupling the DVS to a single-photon (SPAD) front end, or lowering $\theta_{C}$ below 12% with next-generation event sensors.

**S15: Field of View and Motion Sensitivity**

Field of view (FOV) is set by the DVS sensor area (9.6 × 7.2 mm², 640 × 480 pixels at 15 µm pitch), the lens focal length, and the working distance; it scales linearly with working distance and inversely with focal length. The configurations reported in this work span the FOVs and target-plane pixel pitches listed in Table SIII. These choices were dictated by the experimental targets (MNIST, Kanji, bird silhouettes) rather than by any fundamental limit, so meter-scale stand-off scenes (drone or automotive use) or close-range biomedical macroscopy are immediately accessible by changing the optics, with no algorithmic change to the SNN.

**Table SIII.** Field of view and target-plane pixel pitch for the experimental configurations reported in this work. FOV scales linearly with working distance and inversely with lens focal length.

| **Configuration*** | **Lens *f* (mm)** | **Working dist. (cm)** | **FOV at target (cm)** | **Pixel pitch at target (µm)** |
| --- | --- | --- | --- | --- |
| Solid phantom (Figs. 4–6) | ~ 4 | 4 | 6 × 6 | ~ 125 |
| Turbid water (Fig. 7) | ~ 8 | 36 | 32 × 32 | ~ 670 |
| Fog chamber (S2) | ~ 8 | 36 | 32 × 32 | ~ 670 |

**Motion sensitivity.** For a high-contrast target boundary moving at speed v at the target plane, the log-intensity change at a single pixel accumulated during one time-step Δt is approximately *v Δt / p*, where p is the projected pixel pitch at the target plane. A pixel fires an event only when this change exceeds the DVS contrast threshold $\theta_{C}$ ≈ 12 % (S10), so the smallest target speed that produces at least one event per pixel per Δt is $v\_min \approx(\theta\times p) / \Delta t$ —the speed at which the boundary sweeps a fraction $\theta_{C}$ of a pixel during one time bin. Below this, the log-intensity change stays under the hardware threshold and the pixel remains silent. By changing Δt the same pipeline addresses a wide range of target dynamics, as summarized in Table SIV.

**Table SIV.** Minimum resolvable target speed $v\_min \approx(\theta\times p) / \Delta t$ for two target-plane pixel pitches p and three SNN time-steps Δt ($\theta_{C}$≈ 12%).

| **Δt** | **v_min**  **(p ≈ 125 µm)** | **v_min**  **(p ≈ 670 µm)** | **Notes** |
| --- | --- | --- | --- |
| 20 ms | ≈ 1 mm s⁻¹ | ≈ 4 mm s⁻¹ | Time-step used in the present experiments (MNIST-transmission, Kanji-reflection, bird-silhouette). |
| 1 ms | ≈ 15 mm s⁻¹ | ≈ 80 mm s⁻¹ | Achievable without architectural change; sub-second dynamics—faster pedestrians, slow vehicles, biological motion. |
| 100 µs | ≈ 150 mm s⁻¹ | ≈ 0.8 m s⁻¹ | Approaching the DVS per-pixel latency floor of ≈ 220 µs; rapid biological motion, drones, vehicles. |

In practice the target speed in our experiments is set empirically by balancing the display refresh rate (DMD 100 Hz, OLED 120 Hz, E-ink 2.5 Hz), the scattering condition, the illumination level, and the DVS bias settings, rather than by the network time-step alone.

**Reference**

1.	Sekar, S. K. V. *et al.* Solid phantom recipe for diffuse optics in biophotonics applications: a step towards anatomically correct 3D tissue phantoms. *Biomed. Opt. Express* **10**, 2090 (2019).

2.	Naglič, P. *et al.* Optical properties of PlatSil SiliGlass tissue-mimicking phantoms. *Biomed. Opt. Express* **11**, 3753 (2020).

3.	Chen, G., Peng, P., Li, G. & Tian, Y. Training Full Spike Neural Networks via Auxiliary Accumulation Pathway. Preprint at http://arxiv.org/abs/2301.11929 (2023).

4.	Panda, P., Aketi, S. A. & Roy, K. Toward Scalable, Efficient, and Accurate Deep Spiking Neural Networks With Backward Residual Connections, Stochastic Softmax, and Hybridization. *Front. Neurosci.* **14**, (2020).

5.	Kundu, S., Pedram, M. & Beerel, P. A. HIRE-SNN: Harnessing the Inherent Robustness of Energy-Efficient Deep Spiking Neural Networks by Training with Crafted Input Noise. in *2021 IEEE/CVF International Conference on Computer Vision (ICCV)* 5189–5198 (IEEE, Montreal, QC, Canada, 2021). doi:10.1109/ICCV48922.2021.00516.

6.	Yao, M. *et al.* SPIKE-DRIVEN TRANSFORMER V2: META SPIKING NEURAL NETWORK ARCHITECTURE INSPIRING THE DESIGN OF NEXT-GENERATION NEUROMORPHIC CHIPS. (2024).

7.	Horowitz, M. 1.1 Computing’s energy problem (and what we can do about it). in *2014 IEEE International Solid-State Circuits Conference Digest of Technical Papers (ISSCC)* 10–14 (2014). doi:10.1109/ISSCC.2014.6757323.

8.	Neftci, E. O., Mostafa, H. & Zenke, F. Surrogate Gradient Learning in Spiking Neural Networks: Bringing the Power of Gradient-Based Optimization to Spiking Neural Networks. *IEEE Signal Process. Mag.* **36**, 51–63 (2019).

9.	Eshraghian, J. K. *et al.* Training Spiking Neural Networks Using Lessons From Deep Learning. *Proc. IEEE* **111**, 1016–1054 (2023).

10.	Rueckauer, B., Lungu, I.-A., Hu, Y., Pfeiffer, M. & Liu, S.-C. Conversion of Continuous-Valued Deep Networks to Efficient Event-Driven Networks for Image Classification. *Front. Neurosci.* **11**, 682 (2017).

11.	Masquelier, T. & Thorpe, S. J. Unsupervised Learning of Visual Features through Spike Timing Dependent Plasticity. *PLoS Comput. Biol.* **3**, e31 (2007).

12.	Ronneberger, O., Fischer, P. & Brox, T. U-Net: Convolutional Networks for Biomedical Image Segmentation. Preprint at http://arxiv.org/abs/1505.04597 (2015).

13.	Qin, X. *et al.* BASNet: Boundary-Aware Salient Object Detection. in *2019 IEEE/CVF Conference on Computer Vision and Pattern Recognition (CVPR)* 7471–7481 (IEEE, Long Beach, CA, USA, 2019). doi:10.1109/CVPR.2019.00766.

14.	Fang, H., Shrestha, A., Zhao, Z. & Qiu, Q. Exploiting Neuron and Synapse Filter Dynamics in Spatial Temporal Learning of Deep Spiking Neural Network. Preprint at http://arxiv.org/abs/2003.02944 (2020).

15.	Xie, S. & Tu, Z. Holistically-Nested Edge Detection.

16.	de Boer, P.-T., Kroese, D. P., Mannor, S. & Rubinstein, R. Y. A Tutorial on the Cross-Entropy Method. *Ann. Oper. Res.* **134**, 19–67 (2005).

17.	Wang, Z., Simoncelli, E. P. & Bovik, A. C. Multiscale structural similarity for image quality assessment. in *The Thrity-Seventh Asilomar Conference on Signals, Systems & Computers, 2003* vol. 2 1398-1402 Vol.2 (2003).

18.	Mattyus, G., Luo, W. & Urtasun, R. DeepRoadMapper: Extracting Road Topology from Aerial Images. in *2017 IEEE International Conference on Computer Vision (ICCV)* 3458–3466 (IEEE, Venice, 2017). doi:10.1109/ICCV.2017.372.

19.	Ghosh, A. Insect-inspired Spatio-temporal Downsampling of Event-based Input. (2023).

20.	https://docs.prophesee.ai/stable/hw/manuals/biases.html.

21.	Fang, W. *et al.* SpikingJelly: An open-source machine learning infrastructure platform for spike-based intelligence. *Sci. Adv.* **9**, eadi1480 (2023).

22.	https://lava-nc.org/.

23.	Shi, Y., Sheng, W., Fu, Y. & Liu, Y. Overlapping speckle correlation algorithm for high-resolution imaging and tracking of objects in unknown scattering media. *Nat. Commun.* **14**, 7742 (2023).

24.	Jauregui-Sánchez, Y., Penketh, H. & Bertolotti, J. Tracking moving objects through scattering media via speckle correlations. *Nat. Commun.* **13**, 5779 (2022).

25.	Shi, T. *et al.* Computational imaging of moving objects obscured by a random corridor via speckle correlations. *Nat. Commun.* **13**, 4081 (2022).

26.	Xu, Q. *et al.* Imaging and Tracking Through Scattering Medium With Low Bit Depth Speckle. *IEEE Photonics J.* **12**, 1–7 (2020).

27.	Lyu, M., Wang, H., Li, G., Zheng, S. & Situ, G. Learning-based lensless imaging through optically thick scattering media. *Adv. Photonics* **1**, 036002 (2019).

28.	Ma, K., Wang, X., He, S., Zhang, X. & Zhang, Y. Learning to image and track moving objects through scattering media via speckle difference. *Opt. Laser Technol.* **159**, 108925 (2023).

29.	Liu, H. *et al.* Learning-based real-time imaging through dynamic scattering media. *Light Sci. Appl.* **13**, 194 (2024).

30.	Yan, Z. *et al.* Scattering imaging of moving targets based on recurrent neural networks: a fusion of temporal and phase information. *Opt. Express* **32**, 45978–45994 (2024).

31.	Feng, B. Y. *et al.* NeuWS: Neural wavefront shaping for guidestar-free imaging through static and dynamic scattering media. *Sci. Adv.* **9**, eadg4671 (2023).

32.	Kanaev, A. V. *et al.* Imaging through extreme scattering in extended dynamic media. *Opt. Lett.* **43**, 3088 (2018).
